# Measurements of DNA Methylation at Seven Loci in Various Tissues of CD1 Mice

**DOI:** 10.1371/journal.pone.0044585

**Published:** 2012-09-07

**Authors:** Laurynas Daugela, Nicole Nüsgen, Maja Walier, Johannes Oldenburg, Rainer Schwaab, Osman El-Maarri

**Affiliations:** 1 Institute of Experimental Hematology and Transfusion Medicine, University of Bonn, Bonn, Germany; 2 Institute of Medical Biometry, Informatics and Epidemiology (IMBIE), University of Bonn, Bonn, Germany; Brigham and Women’s Hospital, United States of America

## Abstract

In humans, considerable variation in methylation at single loci and repetitive elements in various cells and tissues is observed. Recently, several inter- and intra-tissue correlations for DNA methylation have been reported. To investigate the extent and reproducibility of such correlations, we investigated inter- and intra-tissue methylation correlations among seven different loci in 9 different tissues in a population of 100 healthy seven-week-old CD1 outbred mice. We used a highly quantitative approach to measure methylation levels to high accuracy at two single loci in the alpha-actin and myosine light chain promoters, at three differentially methylated regions of the *Peg3*, *Snrpn* and *Lit1* genes associated with imprinted loci, and at two repetitive elements in the *Line-1* and *IAP-LTR* genes in the various tissues. In this population of mice, methylation at several loci was sex-associated and intra-tissue correlations among the studied loci were observed for brain and spleen. Inter-tissue correlations were rarely observed. To investigate method-dependent experimental variability, we re-analyzed the same spleen and tongue samples using SIRPH and pyrosequencing methods and reconfirmed intra-tissue correlations for spleen and sex-associated correlations for DNA methylation for tongue. When we repeated DNA methylation measurements for a second mouse population raised under similar conditions three months later, we did not detect sex-associated or intra-tissues correlations. Additional studies that examine large numbers of loci may be required to further understand the factors that influence stability of DNA methylation.

## Introduction

DNA methylation plays an essential role in the regulation of gene expression. It modifies the 5-carbon position to yield 5-methylcytosine or 5-hydroxymethylcytosine [1], [2], [3], [4]. Tissue- and cell-specific DNA methylation “fingerprints” have been reported [5], [6]. Deviation from normal levels of methylation are associated with disease and include hyper- or hypomethylation of CpG islands and hypomethylation of repeats found in most tumors in addition to differentially methylated regions associated with imprinting disorders [7], [8]. However, DNA methylation variability has also been reported in healthy individuals. It has been shown that locus-specific variability may be associated with neighboring local polymorphisms [9], [10], may be sex-associated [11], [12], [13], associated with chromosomal aneuploidy [14], or with aging [6], [15], [16], [17], [18]. Apart from genetic factors, DNA methylation levels can also be influenced by various environmental factors [19], [20]. However, which environmental conditions and to what extent they have an effect on DNA methylation remains poorly understood. The lack of affordable, highly sensitive global assays for measuring DNA methylation has also impeded investigation in this area.

Understanding the influence of different environmental conditions on the methylation levels in normal populations and for changes in normal environmental conditions is essential when considering DNA methylation as markers for detecting abnormal physiological states associated with disease. Furthermore, the presence of reliable and reproducible inter-tissue correlations for DNA methylation exclusively associated with healthy or disease conditions might allow the use of methylation, for example in blood cells, as surrogate markers for other less accessible tissues. However, only sparse data on intra- and inter-tissue correlations for DNA methylation exists.

We recently reported some correlations in DNA methylation between adenocarcinomas tumor tissue and peripheral blood cells [21]. Another study reported correlations between buccal and blood cells at four loci including *IGF2R*, *APOC1*, *LEP* and *CRH* [22]. Additionally, at five metastable epialleles, inter-tissue methylation correlations were observed between kidney, liver and brain [23]. Moreover, our group and others have reported sex-associated influence on methylation levels [11], [13]. However, it is not clear if sex-associated and inter-tissue correlations for DNA methylation are consistent across large populations, different environmental conditions and across different genetic loci. Therefore, we undertook this study to investigate intra- and inter-tissue DNA methylation correlations as well as sex-associated correlations and their statistical profiles using two healthy mouse populations.

For one population of mice and a limited number of CpG sites in multiple tissues, our data suggest that sexes differ in methylation levels at several loci and in many tissues. Moreover, intra-tissue correlations were not widely seen and were mainly limited to spleen, brain and bone marrow in our study. Inter-tissue correlations were rarely detected. However, sex-associated differences and intra-tissue correlations for DNA methylation were not found for a separate mouse population. Our data suggest that sex-associated differences and inter- as well as intra-tissue correlations might be affected by several factors including variability in experimental procedures, stochastic variation of epigenetic markers or by unknown environmental factors. Accordingly, our results suggest that more populations and genetic loci will need to be included in future studies to determine if DNA methylation correlations can form the basis of surrogate disease marker diagnosis methods.

## Materials and Methods

### Ethics Statement

Mice were raised and sacrificed according to institutional, national and international guidelines. Animals were raised and sacrificed without any special clinical treatment or invasive procedure. The sacrifice of animals for scientific studies without any clinical handling (prior to killing) is not part of the German animal protection law (paragraphs §8b and §9). This procedure lies within the responsibility of the local animal protection officer at the Institute of Experimental Therapy (University of Bonn, Germany) where the animals were raised. Therefore, no ethics approval was received, as this was not necessary.

### Mouse Populations and Tissue Samples

Two populations of outbreed CD1 mice, each consisting of 100 mice (50 males and 50 females), were included in this study. All mice were about seven weeks old when sacrificed. The two populations were sacrificed roughly three months apart. From the first population, complete organs including brain, skin (ear lobe), tongue muscle, lungs, heart, liver, spleen, kidneys, bone, bone marrow, skeletal muscle, and testis were isolated. From the second population, brain, lung, spleen and tongue muscle were isolated. After the animals were sacrificed by asphyxiation with CO_2_, organs were isolated within minutes and snap frozen in liquid nitrogen before storing at −80°C for later use.

### Genetic Loci Studied

As targets for the methylation analysis we selected three groups of loci. The first group included two single loci with low CpG density: the cytoskeletal α-actin and myosin light chain genes. Both are expressed to different degrees in skeletal, smooth and cardiac muscles. The second group of loci included three genes with differentially methylated regions (DMR) that have high density of CpGs and associated with imprinted genes *Peg* 3 (paternally expressed gene 3), *Snrpn* (small nuclear ribonucleoprotein N) (differential methylated region 1) and *Lit*1 (long intronic transript 1), which are all methylated on the maternal allele and paternally expressed. The third group included repetitive elements that were amplified using degenerate primers targeting high CpG-density regions within the Line-1 (long interspersed element 1) and IAP-LTR (intracisternal A particles-long terminal repeats) sequences. We studied two CpG sites within each region in the first mouse population, while only one CpG was studied in the second population.

### DNA Extraction

The extraction of DNA was performed using the DNeasy blood and tissue kit (Qiagen: Hilden, Germany). Prior to the extraction procedure, the manufacturer-recommended amount of starting tissue was homogenized in a 1.5 ml Eppendorf tube using a single-use plastic hand-held mortar by grinding until no tissue particles were visible. For more mechanically resistant tissues, like bone and cardiac muscle, an electric homogenizer was used. To access the quality and quantity of extracted DNA, concentration of the DNA samples was measured by spectrophotometer and aliquots were loaded on 1% agarose gel to ensure presence of high molecular weight DNA. The A_260nm_/A_280nm_ ratio was >1.8 for all DNA used.

### Bisulfite Treatment and Quantitative Methylation Analysis at Specific CpG Sites

Bisulfite treatment of the DNA was performed according to the EpiTect bisulfite conversion protocol using 1 µg of DNA (Qiagen, Hilden, Germany). Primers used in this study are listed in Table S1, while details of the sequence amplified and the location of the primers can be found in Figure S1. Primers for amplification of the myosine light chain and α-actin were used as described by Oswald *et al*. [24]; *Peg* 3, *Lit*1, Line-1 and IAP-LTR were screened according to Hajkova *et al*. [25], while *Snrpn* was screened according to El-Maarri *et al*. [26]. Amplification of Line-1 and IAP-LTR was done using primers specific for regions within the repeat sequence, thus amplifying all corresponding targets in the mouse genome without being specific for a particular locus (degenerate amplification approach).

Site-specific methylation measurements at two selected CpG sites in every region were carried out using the SIRPH protocol as previously described by El-Maarri *et al*. [27], [28]. We selected the most technically reproducible CpG sites at every locus. The CpGs studied are expected to correlate very well with other CpGs of the locus as previously demonstrated for high-density loci and, especially, when using a highly sensitive method for methylation mapping [29]. SNuPE (single nucleotide primer extension) primers used in this study are listed in Table S1. To minimize variations in the bisulfite treatment conditions, male and female samples for a given tissue were homogeneously distributed on the same plate. Bisulfite amplification and the subsequent SIRPH (SNuPE with IP-RP-HPLC) reaction were performed simultaneously on the same plate in parallel for male and female samples. For PCR and the SIRPH reaction, Hot start FIREPol and TERMIPol from Solis BioDyne (Tartu, Estonia), respectively, were used. To allow valid statistical comparison between male and female subpopulations, both sex samples were always included in the same measurement batches (i.e. same bisulfite treatment, same PCR and same SIRPH reaction). For the pyrosequencing control experiments the same SIRPH primers corresponding to CpG-1 were used as the sequencing primers. Pyrosequencing using the Pyro Gold reagents from (Qiagen, Hilden, Germany) was performed using a PyroMark ID system. The sequence analyzed and the locations of the SIRPH and pyrosequencing primers as well as the positions of the studied CpG sites are shown in Figure S1.

### Statistical Analysis

Analyses were performed using the SAS 9.2 package (SAS Institute Inc., Cary, NC, USA) or the GraphPad Prism version 5. Normal distribution of the data was tested by D’Agostino & Pearson omnibus normality test. Significance of the sex-associated difference in methylation at each locus was calculated by t-test and non-parametric Mann-Whitney test. Pearson and Spearman correlations were performed on the different groups of samples to determine the significance and degree of correlations. All P values were corrected for multiple testing using the Bonferroni method. The significance difference between the levels of methylation at a given locus in different tissues was calculated by the non-parametric Krustal-Wallis test. The power calculation and estimated needed sample size was performed using GPower v.3; the effect size was calculated based on the values in the first population of mice [30].

## Results

### Tissue Specificity of DNA Methylation

**Figure 1:**
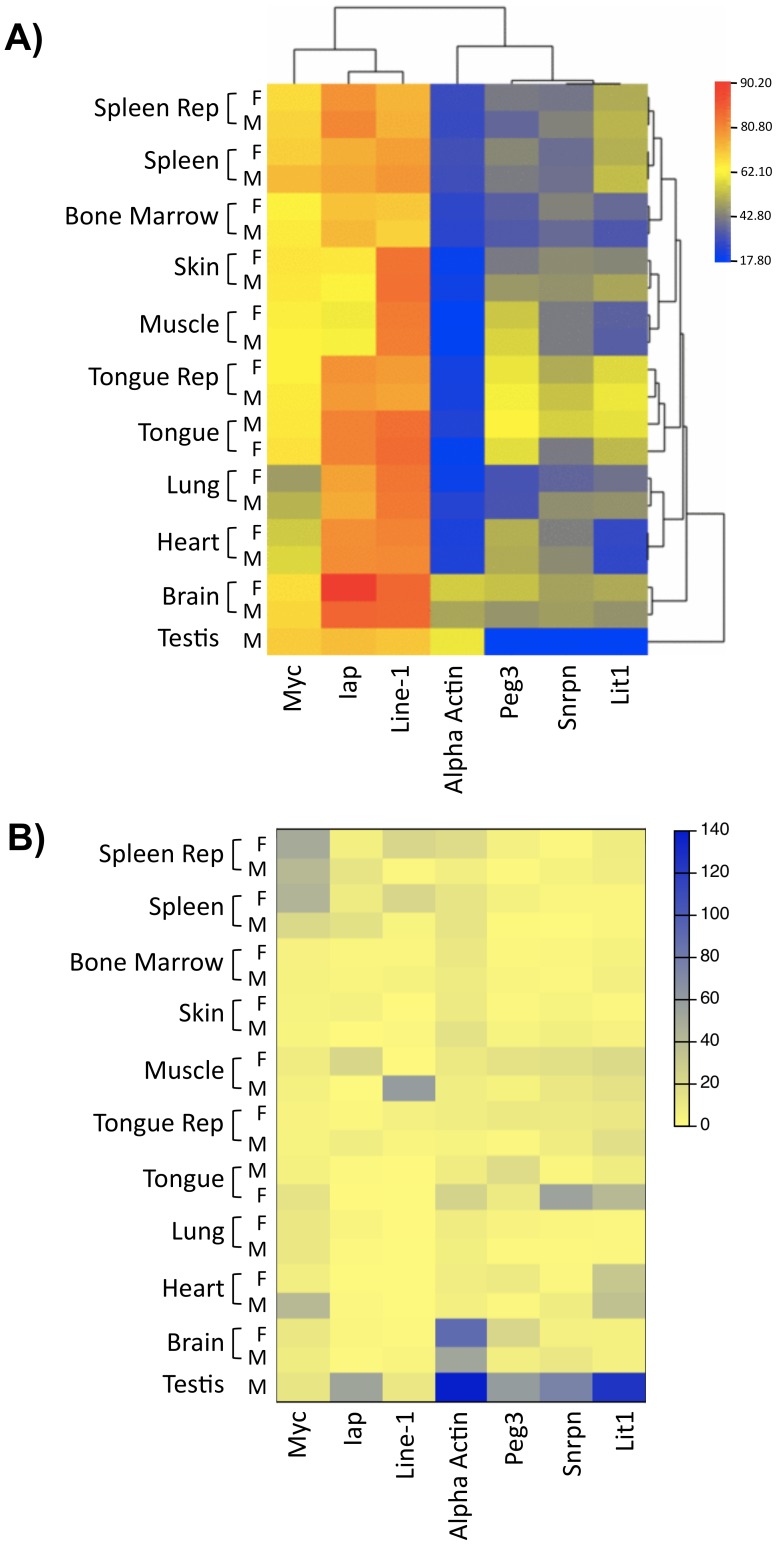
Heat maps of methylation values and variances. A) Heat map and clustering of average methylation values (based on SIRPH results for the CpG-1 and first population of mice). Using the average linkage and Euclidean distance clustering method the average of all methylation values at a given loci/tissue in males (M) and females (F) (separate) are clustered. B) Heat map of variance of methylation values. The variance at each loci and tissue combination in both male and female mice subpopulations is shown. The levels of variances are noticeably similar in both sexes.

Each CpG of the seven studied loci was studied in 8 or 9 different tissues. The tissue specificity of the methylation is evident for each locus (Figure 1A; Table S2). In fact, based on only these seven single CpG methylation markers, correct clustering and classification of the tissues was possible (male and female subpopulations of a given tissue are correctly clustered together). Additionally, non-parametric one-way ANOVA analysis for comparing methylation levels of one loci in different tissues was highly significant for all loci (CpG-1 and CpG-2 for both male and female subpopulations, Krustal-Wallis test, p<0.0001) A relatively greater level of methylation was observed in most tissues at IAP-LTR, Line-1 and *Myc*. The least methylation was measured for α-actin, while imprinted loci exhibited a measured range of 40–60% as expected. Very low methylation levels were observed at the three paternal imprinted genes in testis since male gametes are not methylated at these differentially methylated regions (DMRs).

### Variability of Methylation at a given Locus between Different Individuals

The accurate quantitative methylation measurement provided by the SIRPH reaction (precision of 1–2%) allowed us to study variability at unprecedented high precision at each locus across individuals. All loci showed variable methylation levels in different tissues with variance values ranging from as low as 0.49 for Line-1 in skin to as high as 59.35 for IAP-LTR in muscle in males, and from 0.58% for Line-1 in skin to 90.15 in alpha actin in brain in females (Figure 1B; Table S2). Of note, variability was greatest for testis at imprinted loci. This is due to differences in methylation between germ cells and somatic cells and to the fact that the ratio of these cells in different samples may not be the same (*i.e.*, the tissue is not homogeneous with respect to degree of cellular differentiation). We also noted that the variance of methylation level for α-actin was relatively greater for testis than for all other studied somatic tissues. The degree of variability showed no obvious correlation to a given tissue or to a specific locus. Both sexes showed similar variability at a given locus. This is illustrated by the fact that the locus/tissue combination with the greatest and the least variance in methylation values is the same in sexes. The lowest was at Line-1 in skin, while the highest was at α-actin in brain (excluding testis here) (Figure 1B; Table S2).

### Correlations between Neighboring CpGs in a given Region

For the first mouse population, we analyzed two CpG sites at every region. The detail of the locations and distances between the two studied CpGs are shown in Figure S2. High methylation correlations were seen at most regions and for most tissue with some exceptions. The average Spearman correlation across tissues was the highest for spleen (Rho = 0.7) and the lowest for Tongue (Rho = 0.49); while the correlation average across the loci was the highest for PEG3 (Rho = 0.71) and the lowest for IAP (Rho = 0.46). These correlations clearly show that, in these regions and in the given tissues, correlation at the first CpG site is well correlated with the second CpG site; therefore CpG-1 is a good estimate of the methylation levels of the nearby CpGs for all studied regions. For this reason we proceeded to analyze only one CpG per locus for the second mouse population.

### Sex-association of DNA Methylation

**Figure 2:**
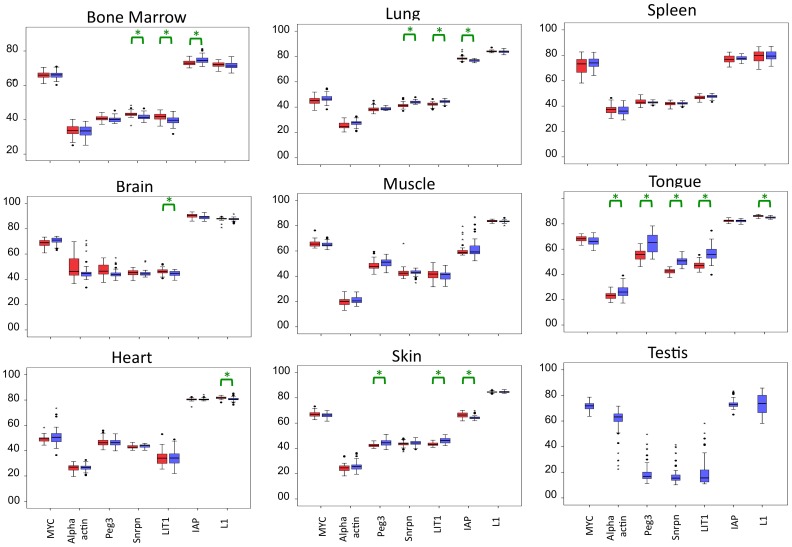
Box plots of methylation values. Box plots represent both male and female individual methylation data at each loci/tissues. Red boxes represent females and blue boxes represent males. Significant t-test (corrected for multiple testing by Bonferoni method) differences between sexes are indicated by a green bracket and a star (for detailed p values see Table S2 and S3).

For the first mouse population, a sex-association comparison of the methylation values at all loci in all tissues allowed 56 comparisons with 26 of them exhibiting statistical significance (Figure 2; Table S2). We note that sex-associated comparisons were performed only on samples included in the same experimental batch (i.e. same bisulfite, same PCR and same SIRPH reaction). It is not valid to compare samples from two different experiments due to differences introduced by different batches of bisulfite treatment and PCR reactions [31]. However, after correction for multiple testing, only 16 of 26 loci remained significant. The sex-association specific effect on methylation at these loci indicates that males were more methylated at some loci, while at other loci females were more methylated. Therefore, sex-associated differences appear to be both locus- and tissue-specific in this population. We also reanalyzed the same data using a non-parametric Mann-Whitney test to exclude bias due to non-normal distribution of some loci. All previously identified correlations were also found to be significant (Table S3).

### Reconfirmation of the Sex-associated Effect on Methylation for Tongue

To establish confidence for the above results, we reanalyzed all seven loci for the same tongue tissue again using the same SIRPH procedure at both CpG-1 and CpG-2 and using an alternative method based on the pyrosequencing methylation assay [32]. Good reproducibility was seen especially between the first SIRPH measurement and the second SIRPH measurement (Table S4). This was true for *MYC*, *PEG3*, *LIT1*, *SNRPN*, *IAP* and *L1*, but was not reproducible for the single non-imprinted loci probably due to the smaller measured differences between sexes. The comparison between the first SIRPH and pyrosequencing methods, however, exhibited good reproducibility at CpG-1 for α-actin, PEG3, and SNRPN. When considering the average of two CpGs in the first SIRPH measurement and the average of all studied CpGs by the pyrosequencing method, we found good reproducibility for α-actin and SNRPN. No comparision was possible for PEG3 and L1 because SIRPH CpG2 data was missing. In summary, a high degree of reproducibility was found between the two SIRPH measurements, but to a less extent between SIRPH and pyrosequencing methods. The later result is due to the intrinsic differences between the two methods and the fact that pyrosequencing includes additional experimental steps that require the isolation of single stranded DNA and, thus, is more prone to artifacts than the SIRPH method. The SIRPH method is more sensitive and has higher resolution for small differences in DNA methylation than pyrosequencing and thus may not be effective for measuring small differences in methylation.

### Intra- and Inter-tissue Correlations of Methylation Levels

#### Inter-tissue correlations of methylation levels

After correction for multiple testing, only three inter-tissue correlations were significant, namely *Lit*1 (muscle)- *Lit*1 (heart) in females at CpG-1, α-actin (spleen)- α-actin (bone marrow) in males at CpG-1 and SNRPN (lung) and PEG3 (Skin) in Females at CpG-1 (Table 1A). Thus, no extensive statistically significant inter-tissue correlation exists between the loci studied in population 1.

**Table 1:** Spearman correlations of significant inter (A) and intra- tissue correlations in the first mice population.

			Males									Females													
			CpG 1			CpG 2			Ave of both			CpG 1			CpG 2					Ave of both					
	Site 1	Site 2	p	p (Cor)	Rho	p	p (Cor)	Rho	p	p (Cor)	Rho	p	p (Cor)	Rho	p		p (Cor)		Rho	p	p (Corr)	Rho			
**A)**	**BM-Aactin**	**Sp-Aactin**	**7,59E−06**	**2,30E−02**	**0,60**	8,16E**−**01	1	0,04	2,10E**−**02	1	0,39	3,56E**−**04	1	0,52	6,46E**−**01		1		0,11	3,79E**−**01	1	0,20			
	**He-LIT1**	**Mu-LIT1**	1,12E**−**03	1	**−**0,47	7,67E**−**01	1	**−**0,04	7,11E**−**02	1	**−**0,27	**7,09E−06**	**2,14E−02**	**−0,62**	7,57E**−**01		1		**−**0,05	5,81E**−**04	1	**−**0,50			
	**Lu-SNRPN**	**SK-PEG3**	1,39E**−**01	1	**−**0,22	7,13E**−**01	1	0,17	3,76E**−**01	1	0,18	**8,37E−06**	**2,53E−02**	**0,59**	2,62E**−**01		1		0,26	1,27E**−**03	1	0,45			
**B)**	**BM-IAP**	**BM-L1**	1,70E**−**01	1	0,20	2,38E**−**03	1	0,44	2,79E**−**03	1	0,43	**3,00E−11**	**9,07E−08**	**0,79**	**7,62E−06**		**2,31E−02**		**0,60**	**2,69E−08**	**8,44E−05**	**0,71**			
	**BM-LIT1**	**BM-IAP**	1,64E**−**01	1	0,22	1,01E**−**01	1	0,26	4,08E**−**02	1	0,32	2,82E**−**01	1	**−**0,16	**1,75E−06**		**5,31E−03**		**0,64**	2,88E**−**02	1	0,32			
	**BM-MYC**	**BM-L1**	5,91E**−**05	1,79E**−**01	0,55	5,68E**−**04	1	0,48	**6,78E−06**	**2,13E−02**	**0,60**	**6,71E−09**	**2,03E−05**	**0,73**	**4,27E−08**		**1,29E−04**		**0,70**	**1,27E−12**	**3,98E−09**	**0,83**			
	**BM-MYC**	**BM-IAP**	7,65E**−**01	1	0,05	9,78E**−**03	1	0,38	7,92E**−**03	1	0,39	**1,05E−07**	**3,18E−04**	**0,68**	**6,47E−06**		**1,96E−02**		**0,60**	**1,40E−08**	**4,39E−05**	**0,71**			
	**Br-Aactin**	**Br-PEG3**	1,19E**−**02	1	0,38	5,05E**−**04	1	0,51	1,20E**−**02	1	0,38	6,07E**−**02	1	0,29	**1,48E−05**		**4,48E−02**		**0,65**	2,24E**−**04	7,01E**−**01	0,58			
	**Br-PEG3**	**Br-SNRPN**	3,83E**−**02	1	0,35	5,29E**−**03	1	0,61	1,50E**−**03	1	0,68	**1,17E−06**	**3,53E−03**	**0,72**	1,63E**−**01		1		0,39	5,12E**−**03	1	0,70			
	**He-MYC**	**He-LIT1**	1,65E**−**05	5,00E**−**02	0,57	**9,26E−06**	**2,80E−02**	**0,59**	**1,36E−06**	**4,26E−03**	**0,63**	8,41E**−**01	1	**−**0,03	4,31E**−**01		1		0,13	2,99E**−**01	1	**−**0,17			
	**He-PEG3**	**He-SNRPN**	5,11E**−**02	1	0,28	5,97E**−**04	1	0,49	1,87E**−**02	1	0,36	1,57E**−**01	1	0,21	**1,58E−05**		**4,77E−02**		**0,63**	4,03E**−**03	1	0,44			
	**SK-PEG3**	**SK-LIT1**	3,47E**−**04	1	0,50	**5,76E−06**	**1,74E−02**	**0,62**	3,03E**−**05	9,52E**−**02	0,58	7,50E**−**04	1	0,46	3,30E**−**02		1		0,30	3,94E**−**03	1	0,40			
	**Sp-IAP**	**Sp-L1**	**1,00E−14**	**3,03E−11**	**0,85**	9,11E**−**04	1	0,46	**1,20E−14**	**3,77E−11**	**0,85**	**1,25E−09**	**3,79E−06**	**0,76**	**5,51E−07**		**1,67E−03**		**0,67**	**5,52E−11**	**1,73E−07**	**0,81**			
	**Sp-MYC**	**Sp-IAP**	**1,92E−16**	**5,82E−13**	**0,88**	4,13E**−**03	1	0,40	**6,30E−12**	**1,97E−08**	**0,80**	**1,45E−11**	**4,37E−08**	**0,81**	5,26E**−**01		1		**−**0,10	**3,38E−12**	**1,06E−08**	**0,83**			
	**Sp-MYC**	**Sp-L1**	**1,14E−11**	**3,46E−08**	**0,79**	**4,68E−15**	**1,41E−11**	**0,85**	**2,79E−15**	**8,75E−12**	**0,85**	**2,06E−17**	**6,23E−14**	**0,90**	**5,13E−12**		**1,55E−08**		**0,82**	**3,07E−16**	**9,62E−13**	**0,89**			
	**Sp-MYC**	**Sp-SNRPN**	2,36E**−**03	1	0,43	1,52E**−**04	4,59E**−**01	0,52	1,33E**−**04	4,18E**−**01	0,52	**4,50E−06**	**1,36E−02**	**0,63**	9,32E**−**03		1		0,38	3,60E**−**05	1,13E**−**01	0,59			
	**Sp-SNRPN**	**Sp-L1**	**8,12E−06**	**2,46E−02**	**0,60**	**3,80E−08**	**1,15E−04**	**0,69**	**1,86E−08**	**5,83E−05**	**0,71**	**3,76E−06**	**1,14E−02**	**0,62**	**1,10E−04**		**3,33E−01**		**0,53**	**1,85E−05**	**5,80E−02**	**0,60**			

Data for two separate CpG sites and their average are shown. Significant p values are in bold.

#### Intra-tissue correlations of methylation levels

Within the same tissue after correction for multiple testing, correlations were still present only for a few tissues, mainly bone marrow, brain, heart and spleen (Table 1B). The main two groups of intra-tissue correlations were seen in bone marrow (for female) and spleen (for both male and female).

### Reconfirming the Intra-tissue Correlation of Methylation in Spleen

Since we observed the largest number of intra-tissue correlations for spleen, we reanalyzed the same spleen tissue samples using two independent experimental methods - SIRPH and pyrosequencing. The second SIRPH measurement exhibited very strong concordance with all intra-spleen correlations observed in the first SIRPH measurement (Figure S3B). However, only the correlation between MYC and SNRPN did not reach significance in the second SIRPH mesurement in males (Figure S3A), while all female correlations were reproducible (Figure S3B). However, the correlations between the two SIRPH measurements for the individual CpGs were not prominent for all loci, with the weakest correlations for the imprinted DMRs. The later may be due to the difficulty in reproducable detemining small methylation differences in the 50% methylation range as often is the case for the DMR regions associated with imprinted loci.

Comparison of the first SIRPH measurement to the pyrosequencing results (when comparing the average of two CpG sites by SIRPH and the average of all studied CpGs in a given pyrosequencing measurement) did not reveal the same intra-spleen correlations except for the L1-SNRPN correlations in males (Figure S4A) and IAP-L1 in females (Figure S4B). Moreover, low reproducibility was observed in the correlation of single CpGs when comparing the same CpGs determined by SIRPH and pyrosequencing. Thus, four out of ten and two out of ten were significantly correlated in males and females, respectively. These data indicate that both SIRPH and pyrosequencing data cannot be directly compared when measuring small incremental differences in DNA methylations.

From the above comparisons, we can also conclude that the correlations between single CpGs, although reproducible when the same measurement method is used, loose significance if methylation of a larger number of CpGs are averaged. This highlights the necessity to consider such data at single CpG resolution when highly accurate methylation measurement methods are used such as SIRPH.

### DNA Methylation Correlations for Mouse Population 2

Next, we used another population of 50 males and 50 females mice to analyze just four tissues, including brain, lung, spleen and tongue, using the SIRPH method. In these four tissues, nine loci showed significant differences between sexes in the first mouse population. In addition, spleen was the only tissue with five observed intra-tissue correlations. However, in the second population, only α-actin in lung was significantly higher methylated in females (data not shown), although this was not the case for the first population. Moreover, no statistically significant intra-tissue correlations were observed for the second population. The sample size needed to replicate the first finding regarding the sex-associated influence on DNA methylation was adequate based on the previously observed effect size observed in for the first population (Table S2).

## Discussion

Examples of highly accurate quantitative assays for methylation analysis are the widely used pyrosequencing-based method and our previously described SIRPH method [27], [28], [32]. The high accuracy in determining the level of methylation at a particular CpG site allows monitoring of naturally occurring non-pathological variability and small fluctuations in the level of methylation that were previously impossible to achieve. Most of the time, these variations are small in their absolute values and may not appreciably affect gene transcription and protein expression levels. However, they may be sensitive markers for indicating a specific environmental exposure or a given cellular state. Moreover, studying methylation with this high precision should enable the detection of relatively weak inter- and intra-tissue correlations between single loci. This represents a specialized field of investigation still in its infancy that, to date has not attracted broad attention. The ability to establish robust correlations for locus-specific methylation is, however, of potentially great importance because not all human tissues are equally accessible for biopsy and genetic analysis for diagnostic purposes. However, if a good correlation exists between a locus in an inaccessible tissue (*e.g.*, brain tissue) and a locus in an accessible tissue (*e.g.*, blood), a diagnostic test could be performed on the correlated accessible tissue to establish the condition of the less accessible tissue. Yet, extensive research is still required to identify and establish inter-tissue correlations for site-specific DNA methylation between both healthy physiological and pathological conditions. In this study, we take a first step in presenting results of an investigation in which we analyzed a small sample of seven genetic loci in nine different tissues in a large population of mice. We have also reanalyzed the same loci, for the tissues that showed the most statistically significant correlations for site-specific DNA methylation in the first population, in a second, equal-sized mouse population. However, if such correlations exist, we were not able to detect them with certainty, possibly due to natural epigenetic variability or due to the effects of unknown environmental factors affecting DNA methylation that we were unaware of and, therefore, could not hold constant for both sample populations of mice. Thus, future studies would likely benefit from including inter-tissue DNA methylation data for more populations to extend our results presented here.

### Sex-associated Correlations on DNA Methylation Across Different Tissues

Previous reports form several groups, including ours, have demonstrated that in human leukocytes [11] and saliva [13], sexes do not exhibit the same DNA methylation levels at all loci. This sex-specific difference was also found to be locus-specific, whereby greater methylation levels were observed in males at certain loci, while at other loci methylation in females was greater [13]. Accordingly, 580 loci out of 27,000 showed differential methylation between male and female saliva samples, but only 8% of the sex-associated loci in saliva were also sex-associated in blood [13]. These results indicate that the sex effect may be either additionally tissue-specific or affected by stochastic variations due to methylation dynamics in living cells.

In accordance with this, we saw no systematic sex-associated bias for all tissues. The sex-associated differences observed for tongue are remarkable; where males are significantly more methylated at most loci (Figure 2; Table S2). Reanalyzing the same set of tissues (first population of mice and using the same SIRPH method) confirmed this tendency and, here again, methylation correlations for imprinted loci also reached significance (Table S4). However, the sex -associated methylation observed for the first population - especially for tongue - was undetected in the second mouse population.

### Intra- and Inter-tissue Correlations

In the repeated analysis of this study, intra and inter-tissue correlations were tested among seven loci in eight tissues. After correction for multiple testing, only one inter-tissue correlation in males (for α-actin between spleen and bone marrow) and two inter-tissue correlations in females (for *Lit-1* between muscle and heart and between SNRPN in lung and PEG3 in skin) were confirmed (Table 1). Thus, in healthy mice, inter-tissue correlation, based on this small number of studied loci, is not widely apparent.

The results presented here from healthy mice populations are seemingly at odds with a number of previous reports that reported inter-tissue correlations [22], [23]. However, a given physiological condition or an underlying disease could explain the de novo appearance of inter-tissue correlations similar to those we recently reported for pancreatic cancer [21]. Future studies of both healthy control populations and of defined disease populations are required for better understanding of the effect of disease on inter-tissue correlations.

In contrast to the absence of widespread statistically significant inter-tissue correlations in other studies, we observed significant numbers of intra-tissue correlations, especially for bone marrow (predominantly in females), and spleen tissues (in both sexes) (Table 1). The fact that many correlations are present in both sexes at several loci is particularly interesting as both subpopulations can be considered as two independent samples. Hence, when both show the same correlations, it is an independent confirmation of the results. Next, we investigated whether the observed intra-tissue correlations are a general phenomenon in second population of healthy mice. To this end, we reanalyzed spleen and brain, which showed the strongest correlations for the first study population. None of the previous correlations were confirmed in the second population (data not shown).

### Possible Reasons for Lack of Congruent Correlations and Sex-associated Effects between Both Sample Populations

The differences in the sex-associated effects and the intra-tissue correlations, between the two mouse populations, clearly indicates variability in methylation levels affecting sex-associated influence and intra-tissue correlations. We suggest three possibilities, of which any one or a combination of the three could explain these variations.

The first possibility is the experimental variability as several factors or artifacts have previously been reported to affect bisulfite based quantitative methylation measurements. DNA extraction and bias in the PCR towards either methylated or unmethylated DNA strands were previously considered [33], [34]. Additionally, different batches of bisulfite treatment and PCR reactions are known to contribute to the variability [31]. This possibility can be excluded from consideration for our study results as we compared samples that were treated in the same batches of bisulfite and PCRs. However, since in this study we have indeed analyzed a very large number of individual samples (1550 different DNA samples and 10,550 PCRs) we cannot completely exclude such an influence. Yet the reproducibility of the results of the same tissue samples when analyzed twice using the same SIRPH technology (namely, intra-tissue correlations for spleen and sex-associated differences for tongue) confirms the technical reproducibility of the measurements and overall quality of the analysis.

The second possibility is the presence of stochastic variation in dynamic methylation in living cells. Variability in methylation in mice has been shown to occur in studied liver and brain tissues and these relatively hypervariable methylation sites were overrepresented in genes related to development, morphogenesis and organogenesis [35], [36]. Li *et al*. also concluded that increasing the methyl donors in the diet increased the number of loci that exhibit variability of methylation [36]. Moreover, stochastic methylation variations have also been recently reported genome-wide for *Arabidopsis thaliana* [37], [38], where the rate of epimutations was estimated at 4.46×10^−4^ per CG site per generation [37]. Such stochastic variations were likely present in both our cohorts of mice and, due to its random nature, will act in different ways for different loci. Thus, sex-associated differences and intra-tissues correlations may not hold for two different populations of mice.

The third possibility explaining the presence of correlations and the much more pronounced sex-associated differences in one population of mice, but not in the other, might be differences in environmental conditions. Presumably, such differences over time could impose pressure to select variations evolving in a more uniform direction at specific loci. This would result in detectable intra-tissue correlations as observed for spleen tissue in one of our cohorts. Both mouse populations were housed at the same facility under similar, standardized conditions, but at two different points in time separated by nearly four months. Although we are not aware of any major environmental differences in the rearing of the two populations, we cannot exclude that these may have substantially contributed to the DNA methylation differences we observed. For example, the first population was raised in August/September, while the second was raised in December/January. Variation in methylation due to uncontrolled seasonal variables, for example, could contribute to the interpopulation differences we found. Another possible environmental variable could stem from CO_2_ inhalation during sacrifice of the mice. This can be associated with activation of several responses in the body of the mouse including activation of anaerobic metabolism resulting in a decrease in alpha-ketoglutarate. The latter is an essential cofactor, together with Fe^++^, for TET enzymes (ten eleven translocation 1, 2 and 3) transforming 5-methyl-cytosine to 5-hydroxymethyl-cytosine into 5-formylcytosine and into 5-carboxylcytosine in sequential oxidations steps [2], [39], [40]. The last products of modified cytosines are believed to be intermediate steps in the demethylation process [40], [41], [42]. Thus, anaerobic metabolism could increase methylation by inhibiting the demethylation process by blocking the formation of the oxidized methyl-cytosine intermediate. Interestingly, the intra-tissue correlations observed by us were mainly in spleen and brain tissues. The highest levels of oxidized methylcytosine intermediates have been found in brain and spleen tissue among the adult tissues studied [39]. Therefore, variability in exposure to CO_2_ (that was kept to a minimum, but was not administered in a measured, fixed dose) and resulting variability in the TET enzyme activities that leads to variability in demethylation among different individual mice could be a possible mechanism underlying the differences in intra-tissue correlations. This hypothesis can be directly tested in future studies.

While we do not have experimental evidence for any of the above possibilities, our data nevertheless clearly demonstrate that methylation levels are not uniform. Instead, they show considerable variability possibly linking environmental conditions with the genome, thus, possibly providing an adaptation mechanism to a given environmental condition. Our results for a very small number of CpGs at seven loci suggest that at least some intra-tissue correlations for locus-specific DNA methylation can be detected using high sensitivity methods as described, and that there is likely an absence of widespread inter-tissue correlations in healthy mouse populations. However, the correlations found in another population of healthy mice were not the same. Therefore, until further studies investigate intra-and inter-tissue correlations in site-specific DNA methylation, results from single sample populations should be interpreted with caution. Accordingly, we recommend verification of correlations for DNA methylation including at least one independent sample population before considering this correlation as indicative of any specific associated condition. An important aspect of our study that has not previously been included in other studies of intra- and inter-tissue correlations of DNA methylation is the focus on comparison of results between large (n = 100) populations, However, limitations of this study include comparison of results for only two sample populations and the relatively small number of genetic loci studied. Future studies that investigate larger numbers of loci and larger number of sample populations for both healthy and pathological population models conditions will be required to establish the extent of correlated DNA methylation sites with an aim towards developing either direct or indirect, surrogate tissue disease-specific diagnostic screening methods.

## Supporting Information

Figure S1 Details of the studied regions. Both wild type (upper panel for each region) and bisulfite sequences (lower panel for each region) are shown. The amplification forward and reverse primers as well as the SNuPE SIRPH primers are labeled with yellow and red respectively. The studied CpG sites in every region are labeled in blue. The pyrosequencing primers correspond to SNuPE primer 1. The CpGs covered by the pyrosequencing are highlighted in red.
(PDF)

Figure S2 Methylation correlation between CpG-1 and CpG-2 for all regions and all tissues of the first population of mice (SIRPH experiments). Heatmaps of both Pearson (right) and Spearman (left) correlation values are shown in part A. In the lower part B the detailed values together with the p value for every correlation are given.
(PDF)

Figure S3 Spearman correlation between the first and the second SIRPH experiment in spleen tissue of the first population of mice for males (A) and females (B). Heatmap of all possible intra-tissue correlations are shown in the upper part (Upper right triangle correspond to the rho values while the lower left correspond to the p values; green boxes correspond to the positions of the significant correlations that was detected by the first SIRPH experiment in the first population of mice as shown in Table S2) and a detailed table of the one to one comparison for every loci in the first SIRPH experiments and the second is shown in the lower part.
(PDF)

Figure S4 Spearman correlation between the first SIRPH and the pyrosequencing of the spleen tissue in the first population of mice. A) data for males and B) female data. Heatmap of all possible intra-tissue correlations are shown in the upper part (Upper right triangle correspond to the rho values while the lower left correspond to the p values; green (for average of CpG-1 and CpG-2 of SIRPH) and blue (for average of all CpGs in pyrosequencing) boxes correspond to the position of the significant correlations that was detected by the first SIRPH experiment in the first population of mice as shown in Table S2) and a detailed table of the one to one comparison for every loci in the first SIRPH experiments and the pyrosequencing is shown in the lower part.
(PDF)

Table S1 List of amplification primers, SIRPH oligos and pyrosequencing primers used in this study.
(XLS)

Table S2 Detailed methylation data for both populations of mice at the first CpG site (SIRPH data). Average (Ave.), median, highest and lowest values, 25% percentile (Q1) and 75% percentile (Q3), standard deviation (SD), variance (Var.) and differences between sexes together with uncorrected and corrected p values of the t-test are given in this table. The values of skewness (Skew.) and kurtosis (Kur.), K2 and P values of the D’Agostino and Pearson omnibus normality test for estimation of normal distribution are also given. Conclusion of the normal distribution test is given in the ND column. The Z-power, the % power and the sample size required to replicate the results are also given. The Blue highlight corresponds to the statistically significant male-female difference after Bonferoni corrections.
(XLS)

Table S3 p values of male-female differences by t-test or the Mann-Whitney test. Blue highlight correspond to the significant differences detected by t-test at CpG-1 in the first mice cohort. Both tests are 100% concordant for the significance.
(XLS)

Table S4 Statistical significance of sex effect on methylation in first cohort tongue tissue. Results of two SIRPH and one pyrosequencing measurements are shown.
(XLS)

## References

[1] Lister R, Pelizzola M, Dowen RH, Hawkins RD, Hon G, et al (2009) Human DNA methylomes at base resolution show widespread epigenomic differences. Nature 462: 315–322. 19829295 10.1038/nature08514 PMC2857523

[2] Tahiliani M, Koh KP, Shen Y, Pastor WA, Bandukwala H, et al (2009) Conversion of 5-methylcytosine to 5-hydroxymethylcytosine in mammalian DNA by MLL partner TET1. Science 324: 930–935. 19372391 10.1126/science.1170116 PMC2715015

[3] Kriaucionis S, Heintz N (2009) The nuclear DNA base 5-hydroxymethylcytosine is present in Purkinje neurons and the brain. Science 324: 929–930. 19372393 10.1126/science.1169786 PMC3263819

[4] Pastor WA, Pape UJ, Huang Y, Henderson HR, Lister R, et al (2011) Genome-wide mapping of 5-hydroxymethylcytosine in embryonic stem cells. Nature 473: 394–397. 21552279 10.1038/nature10102 PMC3124347

[5] Liang P, Song F, Ghosh S, Morien E, Qin M, et al (2011) Genome-wide survey reveals dynamic widespread tissue-specific changes in DNA methylation during development. BMC Genomics 12: 231. 21569359 10.1186/1471-2164-12-231 PMC3118215

[6] Christensen BC, Houseman EA, Marsit CJ, Zheng S, Wrensch MR, et al (2009) Aging and environmental exposures alter tissue-specific DNA methylation dependent upon CpG island context. PLoS Genet 5: e1000602. 19680444 10.1371/journal.pgen.1000602 PMC2718614

[7] Rodriguez-Paredes M, Esteller M (2011) Cancer epigenetics reaches mainstream oncology. Nat Med 17: 330–339. 21386836 10.1038/nm.2305

[8] Bartolomei MS, Ferguson-Smith AC (2011) Mammalian Genomic Imprinting. Cold Spring Harb Perspect Biol. 10.1101/cshperspect.a002592 PMC3119911 21576252

[9] Schalkwyk LC, Meaburn EL, Smith R, Dempster EL, Jeffries AR, et al (2010) Allelic Skewing of DNA Methylation Is Widespread across the Genome. The American Journal of Human Genetics 86: 196–212. 20159110 10.1016/j.ajhg.2010.01.014 PMC2820163

[10] Zhang Y, Rohde C, Reinhardt R, Voelcker-Rehage C, Jeltsch A (2009) Non-imprinted allele-specific DNA methylation on human autosomes. Genome Biology 10: R138. 19958531 10.1186/gb-2009-10-12-r138 PMC2812945

[11] El-Maarri O, Becker T, Junen J, Manzoor SS, Diaz-Lacava A, et al (2007) Gender specific differences in levels of DNA methylation at selected loci from human total blood: a tendency toward higher methylation levels in males. Hum Genet 122: 505–514. 17851693 10.1007/s00439-007-0430-3

[12] El-Maarri O, Walier M, Behne F, van Uum J, Singer H, et al (2011) Methylation at global LINE-1 repeats in human blood are affected by gender but not by age or natural hormone cycles. PLoS ONE 6: e16252. 21311577 10.1371/journal.pone.0016252 PMC3023801

[13] Liu J, Morgan M, Hutchison K, Calhoun VD (2010) A study of the influence of sex on genome wide methylation. PLoS ONE 5: e10028. 20386599 10.1371/journal.pone.0010028 PMC2850313

[14] Kerkel K, Schupf N, Hatta K, Pang D, Salas M, et al (2010) Altered DNA methylation in leukocytes with trisomy 21. PLoS Genet 6: e1001212. 21124956 10.1371/journal.pgen.1001212 PMC2987931

[15] Adkins RM, Thomas F, Tylavsky FA, Krushkal J (2011) Parental ages and levels of DNA methylation in the newborn are correlated. BMC Med Genet 12: 47. 21453505 10.1186/1471-2350-12-47 PMC3078852

[16] Boks MP, Derks EM, Weisenberger DJ, Strengman E, Janson E, et al (2009) The relationship of DNA methylation with age, gender and genotype in twins and healthy controls. PLoS ONE 4: e6767. 19774229 10.1371/journal.pone.0006767 PMC2747671

[17] Fuke C, Shimabukuro M, Petronis A, Sugimoto J, Oda T, et al (2004) Age related changes in 5-methylcytosine content in human peripheral leukocytes and placentas: an HPLC-based study. Ann Hum Genet 68: 196–204. 15180700 10.1046/j.1529-8817.2004.00081.x

[18] Hernandez DG, Nalls MA, Gibbs JR, Arepalli S, van der Brug M, et al (2011) Distinct DNA methylation changes highly correlated with chronological age in the human brain. Human Molecular Genetics 20: 1164–1172. 21216877 10.1093/hmg/ddq561 PMC3043665

[19] Baccarelli A, Wright RO, Bollati V, Tarantini L, Litonjua AA, et al (2009) Rapid DNA methylation changes after exposure to traffic particles. Am J Respir Crit Care Med 179: 572–578. 19136372 10.1164/rccm.200807-1097OC PMC2720123

[20] Bollati V, Baccarelli A, Hou L, Bonzini M, Fustinoni S, et al (2007) Changes in DNA methylation patterns in subjects exposed to low-dose benzene. Cancer Res 67: 876–880. 17283117 10.1158/0008-5472.CAN-06-2995

[21] Dauksa A, Gulbinas A, Barauskas G, Pundzius J, Oldenburg J, et al (2012) Whole blood DNA aberrant methylation in pancreatic adenocarcinoma shows association with the course of the disease: a pilot study. PLoS ONE 7: e37509. 22629410 10.1371/journal.pone.0037509 PMC3358256

[22] Talens RP, Boomsma DI, Tobi EW, Kremer D, Jukema JW, et al (2010) Variation, patterns, and temporal stability of DNA methylation: considerations for epigenetic epidemiology. The FASEB Journal 24: 3135–3144. 20385621 10.1096/fj.09-150490

[23] Waterland RA, Kellermayer R, Laritsky E, Rayco-Solon P, Harris RA, et al (2010) Season of conception in rural gambia affects DNA methylation at putative human metastable epialleles. PLoS Genet 6: e1001252. 21203497 10.1371/journal.pgen.1001252 PMC3009670

[24] Oswald J, Engemann S, Lane N, Mayer W, Olek A, et al (2000) Active demethylation of the paternal genome in the mouse zygote. Curr Biol 10: 475–478. 10801417 10.1016/s0960-9822(00)00448-6

[25] Hajkova P, Erhardt S, Lane N, Haaf T, El-Maarri O, et al (2002) Epigenetic reprogramming in mouse primordial germ cells. Mech Dev 117: 15–23. 12204247 10.1016/s0925-4773(02)00181-8

[26] El-Maarri O, Buiting K, Peery EG, Kroisel PM, Balaban B, et al (2001) Maternal methylation imprints on human chromosome 15 are established during or after fertilization. Nat Genet 27: 341–344. 11242121 10.1038/85927

[27] El-Maarri O (2004) SIRPH analysis: SNuPE with IP-RP-HPLC for quantitative measurements of DNA methylation at specific CpG sites. Methods Mol Biol 287: 195–205. 15273413 10.1385/1-59259-828-5:195

[28] El-Maarri O, Herbiniaux U, Walter J, Oldenburg J (2002) A rapid, quantitative, non-radioactive bisulfite-SNuPE- IP RP HPLC assay for methylation analysis at specific CpG sites. Nucleic Acids Res 30: e25. 11884644 10.1093/nar/30.6.e25 PMC101369

[29] Bock C, Walter J, Paulsen M, Lengauer T (2008) Inter-individual variation of DNA methylation and its implications for large-scale epigenome mapping. Nucleic Acids Research 36: e55–e55. 18413340 10.1093/nar/gkn122 PMC2425484

[30] Buchner A, Erdfelder E (1996) On assumptions of, relations between, and evaluations of some process dissociation measurement models. Conscious Cogn 5: 581–594. 9063617 10.1006/ccog.1996.0034

[31] Irahara N, Nosho K, Baba Y, Shima K, Lindeman NI, et al (2010) Precision of Pyrosequencing Assay to Measure LINE-1 Methylation in Colon Cancer, Normal Colonic Mucosa, and Peripheral Blood Cells. The Journal of Molecular Diagnostics 12: 177–183. 20093385 10.2353/jmoldx.2010.090106 PMC2871724

[32] Tost J, Dunker J, Gut IG (2003) Analysis and quantification of multiple methylation variable positions in CpG islands by Pyrosequencing. Biotechniques 35: 152–156. 12866415 10.2144/03351md02

[33] Warnecke PM, Stirzaker C, Song J, Grunau C, Melki JR, et al (2002) Identification and resolution of artifacts in bisulfite sequencing. Methods 27: 101–107. 12095266 10.1016/s1046-2023(02)00060-9

[34] Harrison J, Stirzaker C, Clark SJ (1998) Cytosines adjacent to methylated CpG sites can be partially resistant to conversion in genomic bisulfite sequencing leading to methylation artifacts. Anal BioChem 1: 129–132. 10.1006/abio.1998.2833 9784198

[35] Feinberg AP, Irizarry RA (2009) Stochastic epigenetic variation as a driving force of development, evolutionary adaptation, and disease. Proceedings of the National Academy of Sciences 107: 1757–1764. 10.1073/pnas.0906183107 PMC2868296 20080672

[36] Li CC, Cropley JE, Cowley MJ, Preiss T, Martin DI, et al (2011) A sustained dietary change increases epigenetic variation in isogenic mice. PLoS Genet 7: e1001380. 21541011 10.1371/journal.pgen.1001380 PMC3080854

[37] Schmitz RJ, Schultz MD, Lewsey MG, O’Malley RC, Urich MA, et al (2011) Transgenerational Epigenetic Instability Is a Source of Novel Methylation Variants. Science 334: 369–373. 21921155 10.1126/science.1212959 PMC3210014

[38] Becker C, Hagmann J, Müller J, Koenig D, Stegle O, et al.. (2011) Spontaneous epigenetic variation in the Arabidopsis thaliana methylome. Nature. 10.1038/nature10555 22057020

[39] Ito S, Shen L, Dai Q, Wu SC, Collins LB, et al (2011) Tet Proteins Can Convert 5-Methylcytosine to 5-Formylcytosine and 5-Carboxylcytosine. Science 333: 1300–1303. 21778364 10.1126/science.1210597 PMC3495246

[40] He YF, Li BZ, Li Z, Liu P, Wang Y, et al (2011) Tet-Mediated Formation of 5-Carboxylcytosine and Its Excision by TDG in Mammalian DNA. Science 333: 1303–1307. 21817016 10.1126/science.1210944 PMC3462231

[41] Maiti A, Drohat AC (2011) Thymine DNA Glycosylase Can Rapidly Excise 5-Formylcytosine and 5-Carboxylcytosine: POTENTIAL IMPLICATIONS FOR ACTIVE DEMETHYLATION OF CpG SITES. Journal of Biological Chemistry 286: 35334–35338. 21862836 10.1074/jbc.C111.284620 PMC3195571

[42] Bhutani N, Burns DM, Blau HM (2011) DNA Demethylation Dynamics. Cell 146: 866–872. 21925312 10.1016/j.cell.2011.08.042 PMC3236603

