# Supplementary material for: Measurements of DNA Methylation at Seven Loci in Various Tissues of CD1 Mice
Source: PLoS One. 2012 Sep 7;7(9):e44585. doi: 10.1371/journal.pone.0044585 (PMC3436786; doi:10.1371/journal.pone.0044585)
Supplement: Figure S1 — Details of the studied regions. Both wild type (upper panel for each region) and bisulfite sequences (lower panel for each region) are shown. The amplification forward and reverse primers as well as the SNuPE SIRPH primers are labeled with yellow and red respectively. The studied CpG sites in every region are labeled in blue. The pyrosequencing primers correspond to SNuPE primer 1. The CpGs covered by the pyrosequencing are highlighted in red. (PDF) [file pone.0044585.s001.pdf]

Myosin light chain

A) Myosin light chain, Original sequence (accession n. X12972):

1141 acatacaca**C G**tggagcaac tcttctgcac aataaatttg gacaggtaaa ggttagaaaa

1201 gtgtgtacca catgctatac accatagtag gggctggaat gaccaaaggg t**CG**ttcttga

mRNA start

1261 ataactt**CG**c acctgggtgg cccaggtccc ctgatta [aga g**CG**aggggtg gtaggatctg

1321 tgttgaggca ccttttcaag tagggagggt ccctggctgt gtgcatggtg ggaggtctct

1381 ggacatttgt atgcattgtc tggtgt**CG**ct gttcagggcc tgtcagtgcc ccagctgggt

1441 ctaggggaag aggctgt**CG**t gtgtcttgtc tctgtggtcc **CG**tttc**CG**gg tgttcacatt

1501 catgtatgtc tgtgtgggtc tgagtgagtg tctgtatatt tatgtctggg tgtttgtatg

1561 cactagtgta tgagtgtctg gatacagcag gcagctgcag tccactcaca gggtcccagg

1621 gtctccagga acaccccaag caca**CG**tggc actccct**CG**a ggactgtcct ccagactagt

1681 tcaacaggct ccttttacaa aactcctaag actagtgtct caggttttac acacccacac

1741 t**CG**caca**CGC GCG**cacacac acacacatgt gtgcacacac acatacatac acactggatt

B) Bisulfite treated sequence with primer annealing sites, bisulfite primer binding sites marked yellow, single nucleotide extension primer binding sites marked red:

1141 atatatata**C G**tggagtaat ttttttgtat aataaatttg gataggtaaa ggttagaaaa

Bisulfite forward primer →

1201 gtgtgtatta tatgttatat attatagtag gggttggaat gattaaaggg t**CG**tttttga

1261 ataattt**CG**t atttgggtgg tttaggtttt ttgattaaga g**CG**aggggtg gtaggatttg

1321 tgttgaggta tttttttaag tagggagggt ttttggttgt gtgtatggtg ggaggttttt

SNuPE primer 1 →

1381 ggatatttgt atgtattgtt tggtgt**CG**tt gtttagggtt tgttagtgtt ttagttgggt

SNuPE primer 2 →

1441 ttaggggaag aggttgt**CG**t gtgttttgtt tttgtggttt **CG**tttt**CG**gg tgtttatatt

1501 tatgtatgtt tgtgtgggtt tgagtgagtg tttgtatatt tatgtttggg tgtttgtatg

1561 tattagtgta tgagtgtttg gatatagtag gtagttgtag tttatttata gggttttagg

← Bisulfite reverse primer

1621 gtttttagga atattttaag tata**CG**tggt attttt**CG**a ggattgtttt ttagattagt

1681 ttaataggtt ttttttataa aatttttaag attagtgttt taggttttat atatttatat

1741 t**CG**tata**CG**t g**CG**tatatat atatatatgt gtgtatatat atatatatat atattggatt

Alpha actin

A) Alpha actin, Original sequence (accession n. M12347:)

361 ggcaggtcag caat**CG**tgtg tccaggtggg cagatctggg gagacctttc aaacaggtaa

421 atcttgggaa gtacagacca g**CG**gtcaaag cagtgacctt tggcccagca cagcccttc**C**

481 **G**tgagccttg gagccagttg ggaggggcag acagctgggg atactctcca tata**CG**gcct

541 ggtc**CG**gtcc tagctacctg ggccagggca gtcctctcct tctttggtca gtgcaggaga

601 cc**CG**gg**CG**gg acccaggctg agaaccagc**C G**aaggaaggg actctagtgc c**CG**acaccca

661 aatatggctt gggaagggca gcaacattct t**CG**ggg**CG**gt gtggggagag ctcc**CG**ggac

mRNA start

721 tatataaaaa cctgtgcaag gggacagg**CG** gtc [aca**CG**ga **CG**taagcctc acttcctacc

781 ct**CG**gcaccc agggcagagt cagagcagca ggtagggtgg aggtggggag ggtgacctgg

841 agacccagca aagaaagcta ttgagccttg gttgtattta gcactgagtt ctggaaattt

901 ctccaaactc acatccagcc cattttgtga ctgggcattt aggatatgcc tgggggtctg

B) Bisulfite treated sequence with primer annealing sites, bisulfite primer binding sites marked yellow, single nucleotide extension primer binding sites marked red:

361 ggtaggttag taat**CG**tgtg tttaggtggg tagatttggg gagatttttt aaataggtaa

421 attttgggaa gtatagatta g**CG**gttaaag tagtgatttt tggtttagta tagttttttt

Bisulfite forward primer →

481 gtgagttttg gagttagttg ggaggggtag atagttgggg atatttttta tata**CG**gttt

541 ggtt**CG**gttt tagttatttg ggttagggta gttttttttt tttttggtta gtgtaggaga

SNuPE primer 1 → SNuPE primer 2 →

601 tt**CG**gg**CG**gg atttaggttg agaattagt**C G**aaggaaggg atttagtgt t**CG**atattta

661 aatatggttt gggaagggta gtaatatttt t**CG**ggg**CG**gt gtggggagag tttt**CG**ggat

721 tatataaaaa tttgtgtaag gggatagg**CG** gttata**CG**ga **CG**taagtttt attttttatt

← Bisulfite reverse primer

781 tt**CG**gtattt agggtagagt tagagtagta ggtagggtgg aggtggggag ggtgatttgg

841 agatttagta aagaaagtta ttgagttttg gttgtattta gtattgagtt ttggaaattt

901 ttttaaattt atatttagtt tattttgtga ttgggtattt aggatatgtt tgggggtttg

Peg3

A) Peg3, Original Sequence (Accession n. AF105262)

2641 **G**tcaactc**CG** tgccttgg**CG** ccaagctgtt gccttgacaa cagcagtctg attggcaggg

2701 tgtgggagg**C G**tggtgaggg cccaaag**CG**g ggaatggggt cttggattgg ttagagagga

mRNA start

2761agctc**CG**cct ctgcagagga ccctgacaag gaggtgtcc**C G**c [agcccttg ctgcaga**CG**c

2821 tggggagtca ggagt**CGCG**g gagga**CG**agc at**CG**gaggag aag**CG**gagag atgtccaccc

2881 tgggctggtg g**CG**c**CG**c**CG**g g**CG**cc**CG**gtt cagtgtgggt gcactagact gc**CG**accctg

2941 gt**CG**ggtgt gtg**CG**tagag tgctgtgctc **CG**gaggtga gtcagc**CG**gc cacctggctg

3001 ctctgcagca tgcaccctct tagatac**CG**t ctgcagagtt cagatggtgt ttggggtg**CG**

3061 ttgc**CGCG**gg ccagggg**CG**g cagaccatat ca**CG**gctccc aagggtaact gacaaggctg

3121 cagactg**CG**c ctt**CG**ggaag gggaatcac ca**CG**gag**CG**g c**CG**tgttgc**C G**cagggatgc

3181 catttaggtg acagggattt aaagtggtat tctataggtc caggcct**CG**g agcctcaggg

B) Bisulfite treated sequence with primer annealing sites, bisulfite primer binding sites marked yellow, single nucleotide extension primer binding sites marked red:

Bisulfite forward primer →

2641 gttaattt**CG** tgttttgg**CG** ttaagttgtt gttttgataa tagtagtttg attggtaggg

2701 tgtgggagg**C G**tggtgaggg tttaaag**CG**g ggaatggggt tttggattgg ttagagagga

2761 agttt**CG**ttt ttgtagagga ttttgataag gaggtgttt**C G**tagtttttg ttgtaga**CG**t

SNuPE primer 1 →

2821 tggggagtta ggagt**CGCG**g gagga**CG**agt at**CG**gaggag aag**CG**gagag atgtttattt

SNuPE primer 2 →

2881 tgggttggtg g**CG**t**CG**t**CG**g g**CG**tt**CG**gtt tagtgtgggt gtattagatt gt**CG**attttg

2941 gt**CG**ggtgt gtg**CG**tagag tgttgtgttt **CG**gaggtga gttagt**CG**gt tatttggttg

3001 ttttgtagta tgtatttttt tagatat**CG**t ttgtagagtt tagatggtgt ttggggtg**CG**

← Bisulfite reverse primer

3061 ttgt**CGCG**gg ttagggg**CG**g tagattatat ta**CG**gttttt aagggtaatt gataaggttg

3121 tagattg**CG**t ttt**CG**ggaag gggaattat ta**CG**gag**CG**g t**CG**tgttgt**C G**tagggatgt

Snrpn

A) Snrpn, Original Sequence (accession n.AF332579)

```
67681 aaaaatctgt gtgatgcttg caatcacttg ggagcaattt ttttaaaaaa ttaaatgtat
67741 ttagtaatag gcaattatat ccattattcc agattgacag tgattttttt tttttaatac
67801 aCGctcaaat ttcCGcagta ggaatgctca agcattcctt ttggtagctg ccttttggca
67861 ggacattcCG gtcagaggga cagagacccc tgcattgCGg caaaaatgtg CGcatgtgca
                                                            mRNA start
67921gccattgcct gggaCGcatg CGtagggagc CGCGCGacaa acctgagcca ttgCGgca [ag
67981 actagCGcag agaggagagg gagcCGgaga tgccagaCGc ttggttctga ggagtgattt
68041 gcaaCGcaat ggagCGagga aggtcagctg ggcttgtgga ttctagtagt gaaagtgcat
```

B) Bisulfite treated sequence with primer annealing sites, bisulfite primer binding sites marked yellow, single nucleotide extension primer binding sites marked red:

```
                        Bisulfite forward primer →
67681 aaaaatttgt gtgatgtttg taattatttg ggagtaattt ttttaaaaaa ttaaatgtat
67741 ttagtaatag gtaattatat ttattatttt agattgatag tgattttttt tttttaatat
67801 aCGtttaaat tttCGtagta ggaatgttta agtatttttt ttggtagttg tttttggta
SNuPE primer 2 →
67861 ggatatttCG gttagaggga tagagatttt tgtattgCGg taaaaatgtg CGtatgtgta
       SNuPE primer 1 →
67921 gttattgttt gggaCGtatg CGtagggagt CGCGCGataa atttgagtta ttgCGgtaag
                                              ← Bisulfite reverse primer
67981 attagCGtag agaggagagg gagtCGgaga tgttagaCGt ttggttttga ggagtgattt
68041 gtaaCGtaat ggagCGagga aggttagttg ggtttgtgga ttttagtagt gaaagtgtat
```

Lit1

A) Lit1, Original Sequence (accession n.AJ271885)

45901 gaacattc**CG** aa**CG**gagccc ctcactctca gcattaaaac agctaccaca taacaaca**CG**

45961 tactccactc actaccttgg tgctggccac ac**CG**ggctac aaagctcagg ggtctccaga

46021 cc**CG**att**CG**g tttcagctcc agtg**CG**ttct gact**CG**gcc**C G**ggtttaga atc**CG**aaggc

46081 ctgagc**CG**gt gtcctaggcc actcaccttg ggact**CG**ac**C G**acct**CG**ggg ctcaaagggc

46141 ctcaagacca cccctgcttc tgtaagcctg ggccacaaag atgggga**CG**t gga**CG**caaaa

46201 ta**CG**agaact gagcca**CG**gc **CG**tgaaa**CG**a ggac**CG**gc**CG** tgaaa**CG**agg ac**CG**agc**CG**t

46261 aactgcaaaa **CG**aata**CG**ga gccactg**CG**g caaaa**CG**aag atggagccca gc**CGCG**aaag

46321 **CGCG**gca**CG**a atcacctctg cttctggc**CG** tgagtgcttg c**CGCG**aggag ggggaggcta

46381 tgatgag**CGC G**gcca**CGCG**g acttg**CG**act tgtgc**CG**tgc tgactcagag aagaaacc**CG**

46441 **CG**ctgagaaa aaaaccatac ctaggagaac catgc**CG**aga aaaagaag**CG** ctgggaacca

46501 agctgaacag aaaagctctc caagtagaat cacacagagg gaaaagaag**C G**tgttgaaga

B) Bisulfite treated sequence with primer annealing sites, bisulfite primer binding sites marked yellow, single nucleotide extension primer binding sites marked red:

45901 gaatattt**CG** aa**CG**gagttt tttattttta gtattaaaat agttattata taataata**CG**

Bisulfite forward primer

45961 tattttattt attattttgg tgttggttat at**CG**ggttat aaagtttagg ggtttttaga

46021 tt**CG**att**CG**g ttttagtttt agtg**CG**tttt gatt**CG**gtt**C G**ggtttaga att**CG**aaggt

46081 ttgagt**CG**gt gttttaggtt atttattttg ggatt**CG**at**C G**attt**CG**ggg tttaaagggt

46141 tttaagatta tttttgtttt tgtaagtttg ggttataaag atgggga**CG**t gga**CG**taaaa

SNuPE primer 1

46201 ta**CG**agaatt gagtta**CG**gt **CG**tgaaa**CG**a ggat**CG**gt**CG** tgaaa**CG**agg at**CG**agt**CG**t

46261 aattgtaaaa **CG**aata**CG**ga gttattg**CG**g taaaa**CG**aag atggagttta gt**CGCG**aaag

SNuPE primer 2

46321 **CGCG**gta**CG**a attattttg tttttggt**CG** tgagtgtttg t**CGCG**aggag ggggaggtta

46381 tgatgag**CGC G**gtta**CGCG**g atttg**CG**att tgtgt**CG**tgt tgatttagag aagaaatt**CG**

46441 **CG**ttgagaaa aaaattatat ttaggagaat tatgt**CG**aga aaaagaag**CG** ttgggaatta

Bisulfite reverse primer

46501 agttgaatag aaaagttttt taagtagaat tatatagagg gaaaagaag**C G**tgttgaaga

46561 aaaattgaga gaatttagta ggttaaaaaa aaatgttgag aagttaagtg gat**CGCG**tta

Line-1

A) Line-1 Original Sequence (accession n. D84391)

421 t**CG**ccatctt ggtc**CG**ggac c**CG**c**CG**aact taggaaatta gtctgaacag gtgagagggt
481 g**CG**ccagaga acctgacagc ctctggaaca ggcagaagca cagaggggct gaggcagcac
541 cctgagtggg c**CG**ggacag c**CG**gccacct tc**CG**gac**CG**g aggacaggtg cc**CG**cc**CG**gc
601 tggggagg**CG** acctaagcca cagcagcag**C G**gt**CG**ccatc ttggtc**CG**gg acc**CG**c**CG**aa
661 cttaggaaat tagtctgaac aggtgagagg gtg**CG**ccaga gaacctgaca gcttctggaa
721 cagg**CG**gaag cacagagg**CG** ctgaggcagc accctg**CG**tg ggc**CG**ggac agc**CG**gccac
781 cttc**CG**gacc agaggacagg tgcccacc**CG** gctggggagg **CG**gcctaagc cacagcagca
841 g**CG**gt**CG**cca tcttggtc**CG** ggacc**CG**c**CG** aacttaggaa attagtctga acaggtgaga
901 gggtg**CG**cca gagaacctga cagcttctgg aacaggcaga agcacagagg ggctgaggca
961 gcaccctgtg tgggc**CG**ggg acagc**CG**gcc accttc**CG**ga c**CG**gaggaca ggtgcccacc
1021 **CG**gctgggga gg**CG**gcctaa gccacagcag cag**CG**gt**CG**c catcttggtc c**CG**ggactcc
1081 aaggaactta ggaatttagt ctgcttaagt gagagtctgt accacctggg aactgccaaa
1141 gcaacacagt gtctgagaaa ggtcctgttt tgggccttct tctt**CG**gcca ggaggaggtc

B) Bisulfite treated sequence with primer annealing sites, bisulfite primer binding sites marked yellow, single nucleotide extension primer binding sites marked red:

421 t**CG**ttatttt ggtt**CG**ggat t**CG**t**CG**aatt taggaaatta gtttgaatag gtgagagggt

Bisulfite forward primer →

481 g**CG**ttagaga atttgatagt ttttggaata ggtagaagta tagaggggtt gaggtagtat
541 tttgagtggg t**CG**ggatag t**CG**gttattt tt**CG**gat**CG**g aggataggtg tt**CG**tt**CG**gt
601 tggggagg**CG** atttaagtta tagtagtag**C G**gt**CG**ttatt ttggtt**CG**gg att**CG**t**CG**aa
661 tttaggaaat tagtttgaat aggtgagagg gtg**CG**ttaga gaatttgata gtttttggaa
721 tagg**CG**gaag tatagagg**CG** ttgaggtagt attttg**CG**tg ggt**CG**ggat agt**CG**gttat
781 tttt**CG**gatt agaggatagg tgtttatt**CG** gttggggagg **CG**gtttaagt tatagtagta
841 g**CG**gt**CG**tta ttttggtt**CG** ggatt**CG**t**CG** aatttaggaa attagtttga ataggtgaga
901 gggtg**CG**tta gagaatttga tagtttttgg aataggtaga agtatagagg ggttgaggta

SNuPE primer 1 →　　　　SNuPE primer 2 →

961 gtattttgtg tgggt**CG**ggg atagt**CG**gtt attttt**CG**ga t**CG**gaggata ggtgtttatt
1021 **CG**gttgggga gg**CG**gtttaa gttatagtag tag**CG**gt**CG**t tattttggtt t**CG**ggatttt

← Bisulfite reverse primer

1081 aaggaattta ggaatttagt ttgtttaagt gagagtttgt attatttggg aattgttaaa
1141 gtaatatagt gtttgagaaa ggttttgttt tgggtttttt tttt**CG**gtta ggaggaggtt

IAP (LTR)

A) Long Tandem repeat of IAP, Original Sequence (accession n. M17551)

1 tgttgggagc **CGCG**cccaca tt**CG**c**CG**tta caagatgg**CG** ctgacagctg tgttctaagt
61 ggtaaacaaa taatctg**CG**c atatgc**CG**ag ggtggttctc tactccatgt gctctgcctt
121 ccc**CG**tga**CG** tcaact**CG**gc **CG**atgggctg cagccaatca gggagtgaca **CG**tcctagg**C**
181 **G**aaatataac tctcctaaaa aaggga**CG**gg gttt**CG**tttt ctctctctct tgcttcttac
241 actcttgctc ctgaagatgt aagcaataaa gttttgc**CG**c agaagattct ggtctgtggt
301 gttcttcctg gc**CG**gg**CG**tg agaa**CGCG**tc taataacaat tggtgac**CG**a attc**CG**gga**C**
361 **G**agaaaaaac t**CG**ggactgg **CG**caaggaag atccctcatt ccagaaccag aactg**CG**ggt

B) Bisulfite treated sequence with primer annealing sites, bisulfite primer binding sites marked yellow, single nucleotide extension primer binding sites marked red:

Bisulfite forward primer →
1 tgttgggagt **CGCG**tttata tt**CG**t**CG**tta taagatgg**CG** ttgatagttg tgttttaagt
61 ggtaaataaa taatttg**CG**t atatgt**CG**ag ggtggttttt tattttatgt gttttgtttt
SNuPE primer 1 →
121 ttt**CG**tga**CG** ttaatt**CG**gt **CG**atgggttg tagttaatta gggagtgata **CG**ttttagg**C**
SNuPE primer 2 →
181 **G**aaatataat ttttttaaaa aaggga**CG**gg gttt**CG**tttt tttttttttt tgttttttat
← Bisulfite reverse primer
241 atttttgttt ttgaagatgt aagtaataaa gttttgt**CG**t agaagatttt ggtttgtggt
301 gttttttttg gt**CG**gg**CG**tg agaa**CGCG**tt taataataat tggtgat**CG**a attt**CG**ggat
361 gagaaaaaat t**CG**gattgg **CG**taaggaag atttttatt ttagaattag aattg**CG**ggt
