# Supplementary material for: Measurements of DNA Methylation at Seven Loci in Various Tissues of CD1 Mice
Source: PLoS One. 2012 Sep 7;7(9):e44585. doi: 10.1371/journal.pone.0044585 (PMC3436786; doi:10.1371/journal.pone.0044585)
Supplement: Figure S2 — Methylation correlation between CpG-1 and CpG-2 for all regions and all tissues of the first population of mice (SIRPH experiments). Heatmaps of both Pearson (right) and Spearman (left) correlation values are shown in part A. In the lower part B the detailed values together with the p value for every correlation are given. (PDF) [file pone.0044585.s002.pdf]

A)

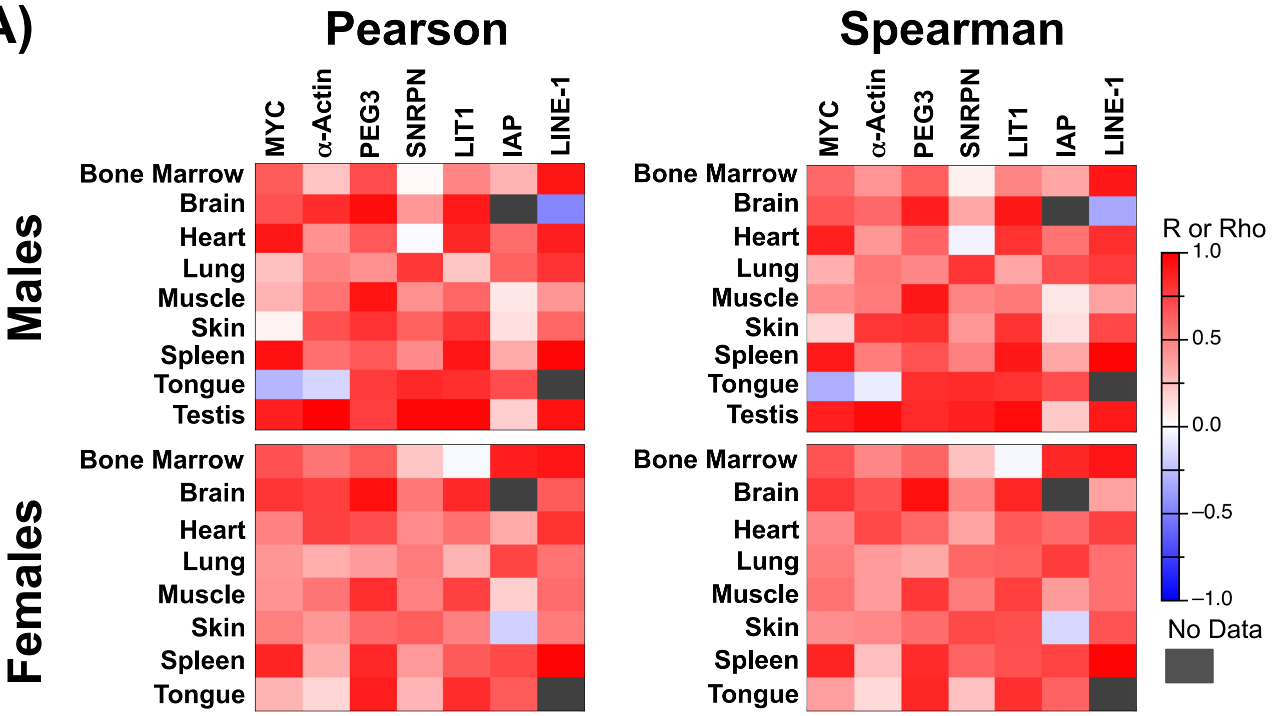

B)

Pearson / Spearman

			Myc	A-Actin	PEG3	SNRPN	LIT1	IAP	L1		Myc	A-Actin	PEG3	SNRPN	LIT1	IAP	L1
Males	**Bone Marrow**	ρ	**0,59**	**0,42**	**0,63**	**0,06**	**0,48**	**0,35**	**0,91**	**r**	**0,64**	**0,23**	**0,71**	**0,03**	**0,48**	**0,30**	**0,92**
		**p**	1,01E-05	3,70E-03	6,96E-05	7,81E-01	1,51E-03	1,58E-02	1,75E-19	**p**	8,38E-07	1,18E-01	3,20E-06	9,03E-01	1,39E-03	4,51E-02	6,43E-21
	**Brain**	ρ	**0,67**	**0,59**	**0,88**	**0,35**	**0,91**		**-0,35**	**r**	**0,69**	**0,83**	**0,95**	**0,41**	**0,91**		**-0,48**
		**p**	1,07E-06	1,84E-05	8,11E-15	1,35E-01	3,70E-18		1,72E-02	**p**	3,96E-07	3,01E-12	4,37E-22	7,42E-02	8,95E-18		6,74E-04
	**Heart**	ρ	**0,88**	**0,41**	**0,61**	**-0,05**	**0,80**	**0,55**	**0,82**	**r**	**0,91**	**0,43**	**0,65**	**-0,02**	**0,85**	**0,57**	**0,88**
		**p**	3,69E-17	1,30E-01	9,85E-06	7,47E-01	4,34E-12	3,46E-05	3,77E-13	**p**	5,27E-20	1,07E-01	1,51E-06	9,08E-01	1,26E-14	1,28E-05	1,73E-17
	**Lung**	ρ	**0,32**	**0,54**	**0,47**	**0,80**	**0,35**	**0,69**	**0,77**	**r**	**0,25**	**0,49**	**0,43**	**0,78**	**0,23**	**0,62**	**0,80**
		**p**	5,04E-02	8,00E-03	3,02E-02	1,38E-02	6,08E-02	2,58E-08	5,99E-09	**p**	1,27E-01	1,69E-02	4,89E-02	1,27E-02	2,40E-01	1,25E-06	5,33E-10
	**Muscle**	ρ	**0,45**	**0,52**	**0,91**	**0,48**	**0,53**	**0,10**	**0,37**	**r**	**0,30**	**0,55**	**0,92**	**0,43**	**0,60**	**0,10**	**0,41**
		**p**	4,80E-03	1,12E-02	4,64E-11	2,55E-03	1,43E-04	5,14E-01	9,45E-03	**p**	7,04E-02	6,59E-03	6,46E-12	7,27E-03	8,03E-06	5,16E-01	3,38E-03
	**Skin**	ρ	**0,16**	**0,79**	**0,81**	**0,41**	**0,80**	**0,13**	**0,72**	**r**	**0,05**	**0,69**	**0,80**	**0,62**	**0,80**	**0,13**	**0,60**
		**p**	2,65E-01	8,78E-03	9,06E-12	9,27E-02	2,41E-11	4,01E-01	9,27E-09	**p**	7,22E-01	2,87E-02	2,85E-11	5,82E-03	4,06E-11	4,06E-01	7,56E-06
	**Spleen**	ρ	**0,91**	**0,51**	**0,67**	**0,50**	**0,91**	**0,35**	**0,98**	**r**	**0,93**	**0,56**	**0,65**	**0,46**	**0,92**	**0,33**	**0,98**
		**p**	1,14E-19	1,04E-03	1,11E-07	3,22E-04	1,47E-19	1,46E-02	7,62E-37	**p**	4,61E-23	2,27E-04	4,07E-07	1,02E-03	1,27E-20	1,88E-02	3,27E-36
	**Tongue**	ρ	**-0,32**	**-0,07**	**0,82**	**0,83**	**0,81**	**0,70**		**r**	**-0,28**	**-0,16**	**0,77**	**0,85**	**0,82**	**0,70**	
		**p**	4,30E-02	8,11E-01	1,33E-01	6,27E-10	2,41E-11	4,04E-08		**p**	7,14E-02	5,95E-01	1,29E-01	1,46E-10	5,90E-12	5,30E-08	
	**Testis**	ρ	**0,88**	**0,96**	**0,84**	**0,88**	**0,96**	**0,21**	**0,91**	**r**	**0,88**	**0,99**	**0,75**	**0,98**	**0,98**	**0,19**	**0,93**
		**p**	1,25E-15	3,18E-28	4,48E-14	4,33E-17	1,20E-06	1,56E-01	1,37E-19	**p**	1,01E-15	0,00E+00	5,08E-10	2,43E-34	2,74E-08	1,97E-01	2,20E-22
Females	**Bone Marrow**	ρ	**0,67**	**0,47**	**0,61**	**0,25**	**-0,03**	**0,85**	**0,92**	**r**	**0,68**	**0,55**	**0,64**	**0,22**	**-0,03**	**0,89**	**0,92**
		**p**	1,45E-07	6,91E-04	1,13E-04	2,07E-01	8,35E-01	9,26E-15	8,05E-20	**p**	8,85E-08	6,11E-05	2,92E-05	2,56E-01	8,65E-01	2,47E-17	9,21E-20
	**Brain**	ρ	**0,79**	**0,68**	**0,94**	**0,48**	**0,86**		**0,37**	**r**	**0,79**	**0,76**	**0,94**	**0,53**	**0,84**		**0,64**
		**p**	1,51E-10	2,65E-07	3,90E-18	7,23E-02	1,69E-14		9,76E-03	**p**	1,72E-10	1,85E-09	2,35E-17	4,01E-02	1,89E-13		1,01E-06
	**Heart**	ρ	**0,47**	**0,72**	**0,60**	**0,36**	**0,65**	**0,58**	**0,75**	**r**	**0,49**	**0,75**	**0,70**	**0,46**	**0,56**	**0,34**	**0,80**
		**p**	1,55E-03	8,81E-02	3,03E-05	1,22E-02	5,74E-07	8,31E-06	8,70E-10	**p**	9,18E-04	5,27E-02	2,48E-07	1,25E-03	4,00E-05	1,71E-02	7,34E-12
	**Lung**	ρ	**0,52**	**0,39**	**0,34**	**0,60**	**0,62**	**0,76**	**0,56**	**r**	**0,41**	**0,32**	**0,40**	**0,52**	**0,30**	**0,73**	**0,55**
		**p**	2,46E-04	1,44E-02	3,35E-02	4,33E-03	6,62E-06	1,01E-08	2,13E-05	**p**	4,31E-03	4,90E-02	1,25E-02	1,49E-02	5,17E-02	8,14E-08	3,58E-05
	**Muscle**	ρ	**0,55**	**0,39**	**0,78**	**0,51**	**0,76**	**0,40**	**0,56**	**r**	**0,43**	**0,54**	**0,82**	**0,50**	**0,75**	**0,19**	**0,58**
		**p**	1,23E-03	6,29E-02	2,83E-08	3,10E-04	1,10E-09	4,65E-03	2,64E-05	**p**	1,69E-02	7,46E-03	1,57E-09	5,37E-04	1,95E-09	1,99E-01	1,51E-05
	**Skin**	ρ	**0,44**	**0,47**	**0,58**	**0,72**	**0,70**	**-0,16**	**0,68**	**r**	**0,50**	**0,41**	**0,59**	**0,64**	**0,50**	**-0,18**	**0,52**
		**p**	1,23E-03	2,64E-02	1,24E-05	3,98E-03	2,01E-08	2,80E-01	8,59E-08	**p**	2,16E-04	5,97E-02	5,98E-06	1,37E-02	2,47E-04	2,07E-01	1,29E-04
	**Spleen**	ρ	**0,86**	**0,25**	**0,83**	**0,61**	**0,69**	**0,74**	**0,98**	**r**	**0,86**	**0,33**	**0,85**	**0,40**	**0,65**	**0,72**	**0,99**
		**p**	2,09E-14	2,46E-01	1,15E-10	5,75E-06	6,04E-08	6,59E-09	2,32E-35	**p**	2,72E-14	1,28E-01	2,22E-11	5,31E-03	6,21E-07	3,38E-08	5,91E-37
	**Tongue**	ρ	**0,38**	**0,15**	**0,85**	**0,24**	**0,82**	**0,61**		**r**	**0,29**	**0,16**	**0,89**	**0,29**	**0,83**	**0,64**	
		**p**	1,43E-02	4,14E-01	3,33E-02	1,10E-01	4,89E-13	2,72E-06		**p**	6,01E-02	3,85E-01	1,80E-02	5,39E-02	1,26E-13	6,55E-07	
