# Supplementary material for: Measurements of DNA Methylation at Seven Loci in Various Tissues of CD1 Mice
Source: PLoS One. 2012 Sep 7;7(9):e44585. doi: 10.1371/journal.pone.0044585 (PMC3436786; doi:10.1371/journal.pone.0044585)
Supplement: Figure S3 — Spearman correlation between the first and the second SIRPH experiment in spleen tissue of the first population of mice for males (A) and females (B). Heatmap of all possible intra-tissue correlations are shown in the upper part (Upper right triangle correspond to the rho values while the lower left correspond to the p values; green boxes correspond to the positions of the significant correlations that was detected by the first SIRPH experiment in the first population of mice as shown in Table S2) and a detailed table of the one to one comparison for every loci in the first SIRPH experiments and the second is shown in the lower part. (PDF) [file pone.0044585.s003.pdf]

A) Males: mice group 1 spleen SIRPH 1 and 2 correlations

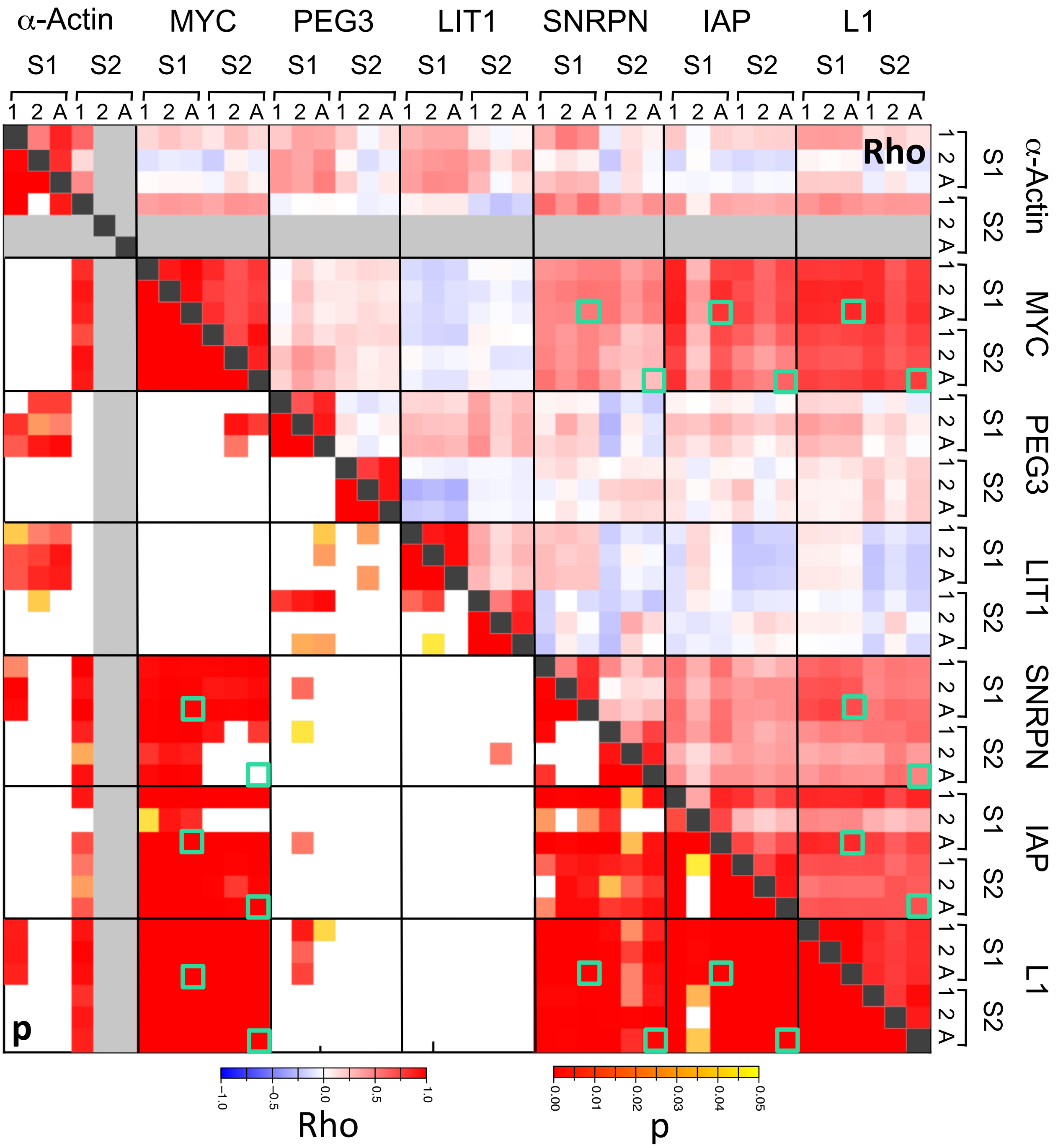

S1 and S2: First and second SIRPH experiment 1, 2 and A: CpG-1, CpG-2 and average of both

	α–Actin-1	α–Actin-2	MYC-1	MYC-2	PEG3-1	PEG3-2	LIT1-1	LIT1-2	SNRPN-1	SNRPN-2	IAP-1	IAP-2	L1-1	L1-2
Rho	0,60		0,84	0,70	-0,05	-0,01	0,33	0,16	0,46	0,15	0,81	0,19	0,84	0,77
P	7,52E-06		3,62E-14	4,21E-08	7,25E-01	9,19E-01	2,03E-02	2,64E-01	1,32E-03	3,10E-01	4,56E-12	2,02E-01	1,62E-13	1,28E-10

B) Females: mice cohort 1 spleen SIRPH 1 and 2 correlations

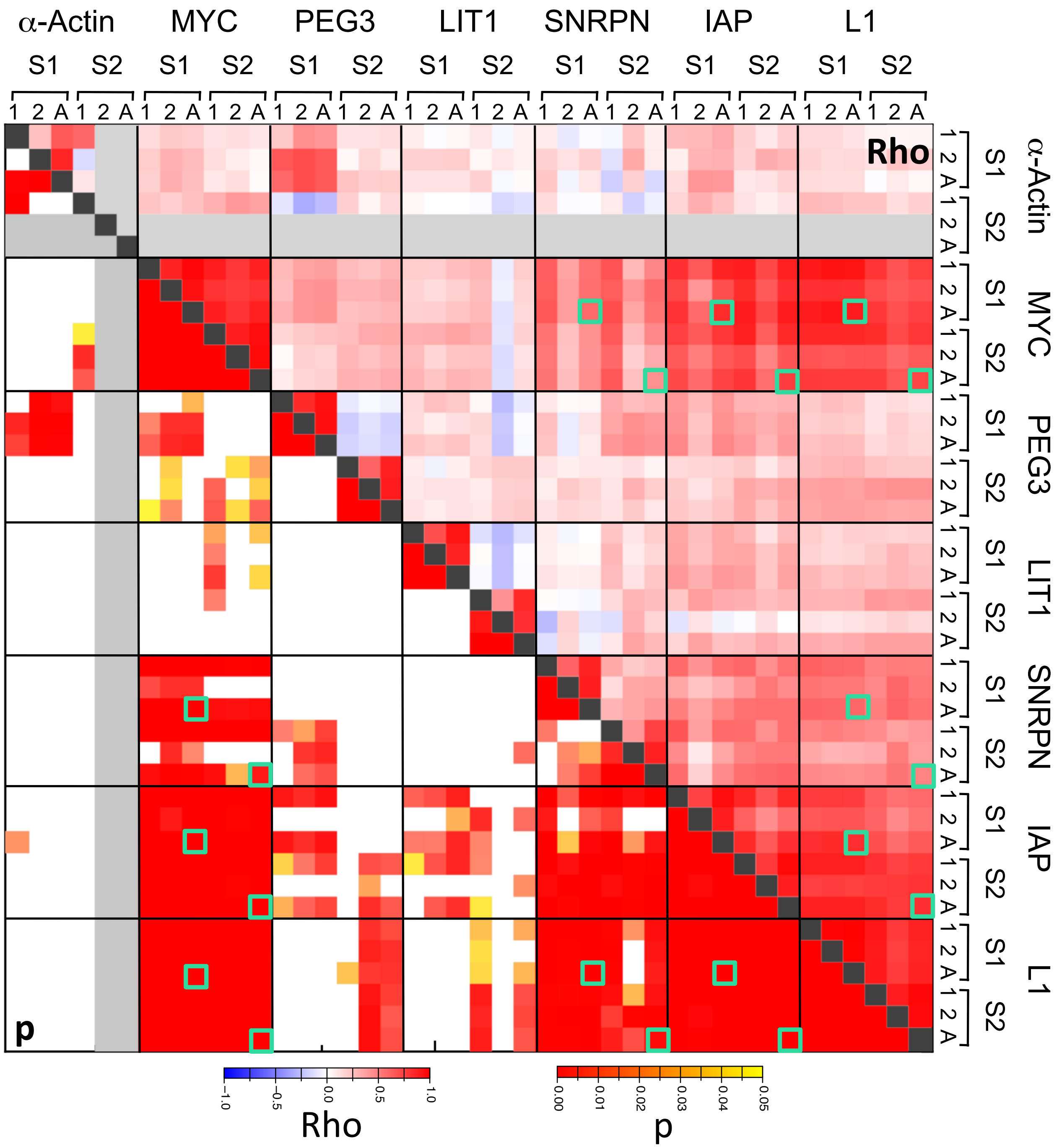

S1 and S2: First and second SIRPH experiment 1, 2 and A: CpG-1, CpG-2 and average of both

	α–Actin-1	α–Actin-2	MYC-1	MYC-2	PEG3-1	PEG3-2	LIT1-1	LIT1-2	SNRPN-1	SNRPN-2	IAP-1	IAP-2	L1-1	L1-2
**Rho**	0,60		0,89	0,77	-0,09	-0,11	-0,13	-0,25	0,34	0,35	0,85	0,45	0,91	0,76
**P**	8,72E-05		1,61E-16	3,86E-10	5,54E-01	5,02E-01	3,87E-01	1,08E-01	2,37E-02	2,72E-02	4,39E-14	2,05E-03	3,16E-18	8,52E-10
