# Supplementary material for: Measurements of DNA Methylation at Seven Loci in Various Tissues of CD1 Mice
Source: PLoS One. 2012 Sep 7;7(9):e44585. doi: 10.1371/journal.pone.0044585 (PMC3436786; doi:10.1371/journal.pone.0044585)
Supplement: Figure S4 — Spearman correlation between the first SIRPH and the pyrosequencing of the spleen tissue in the first population of mice. A) data for males and B) female data. Heatmap of all possible intra-tissue correlations are shown in the upper part (Upper right triangle correspond to the rho values while the lower left correspond to the p values; green (for average of CpG-1 and CpG-2 of SIRPH) and blue (for average of all CpGs in pyrosequencing) boxes correspond to the position of the significant correlations that was detected by the first SIRPH experiment in the first population of mice as shown in Table S2) and a detailed table of the one to one comparison for every loci in the first SIRPH experiments and the pyrosequencing is shown in the lower part. (PDF) [file pone.0044585.s004.pdf]

A) Male first mice cohort spleen SIRPH vs. Pyro.

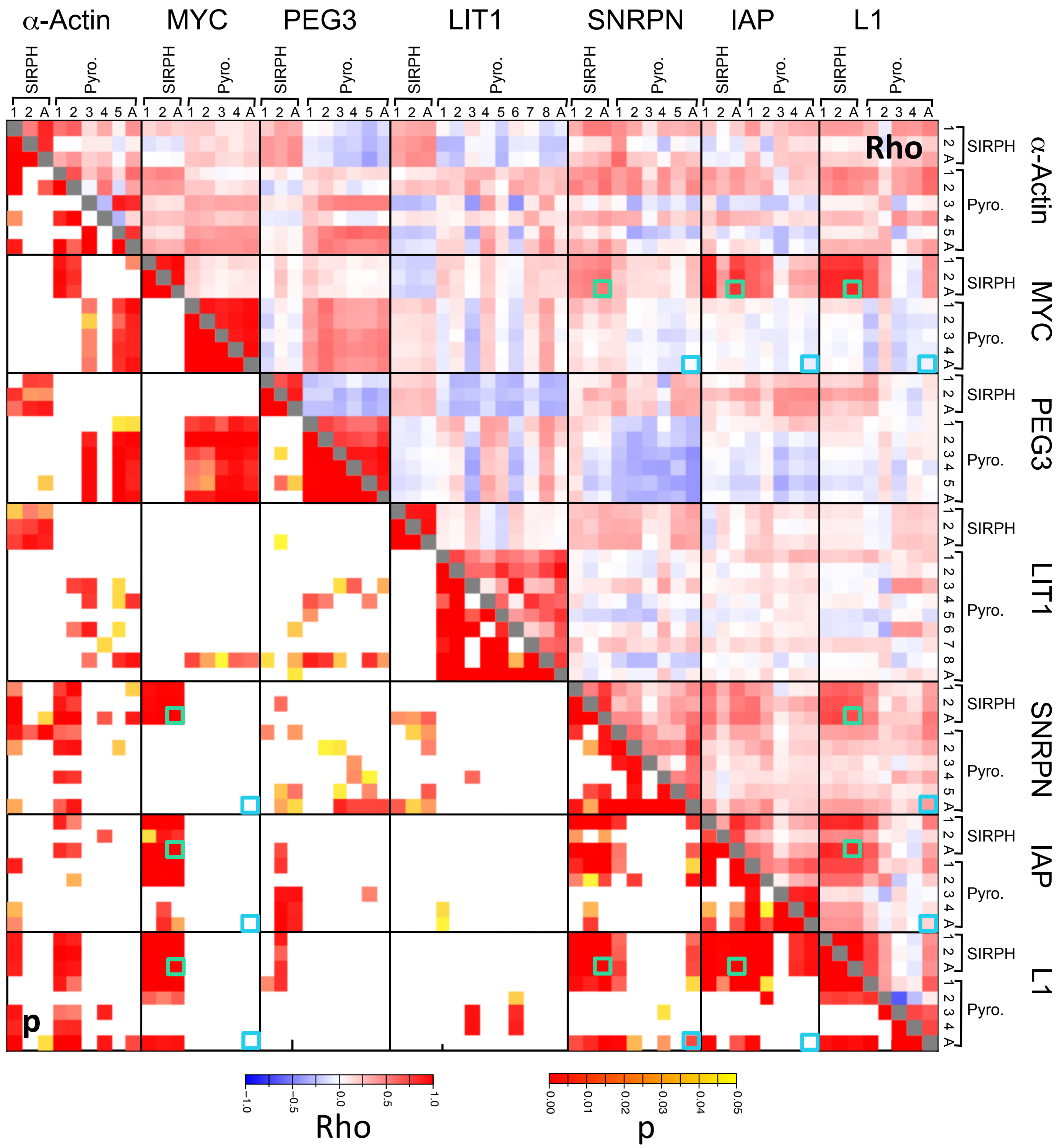

1, 2, …, A: CpG-1, CpG-2, etc, and average;
note that the CpG numbers for SIRPH and Pyro. do not necessarily correspond to the same CpG.

	α–Actin	α–Actin	MYC	MYC	PEG3	LIT1	SNRPN	IAP	IAP	L1
**SIRPH**	1	2	1	2	1	1	1	1	2	1
**Pyro**	1	2	1	3	1	1	1	1	3	1
**Rho**	0,55	0,01	0,21	0,05	-0,25	0,10	0,41	0,65	0,02	0,74
**P**	2,08E-04	9,67E-01	1,73E-01	7,69E-01	1,17E-01	5,34E-01	9,48E-03	1,21E-06	9,13E-01	8,96E-09

B) Female first mice cohort spleen SIRPH vs. Pyro.

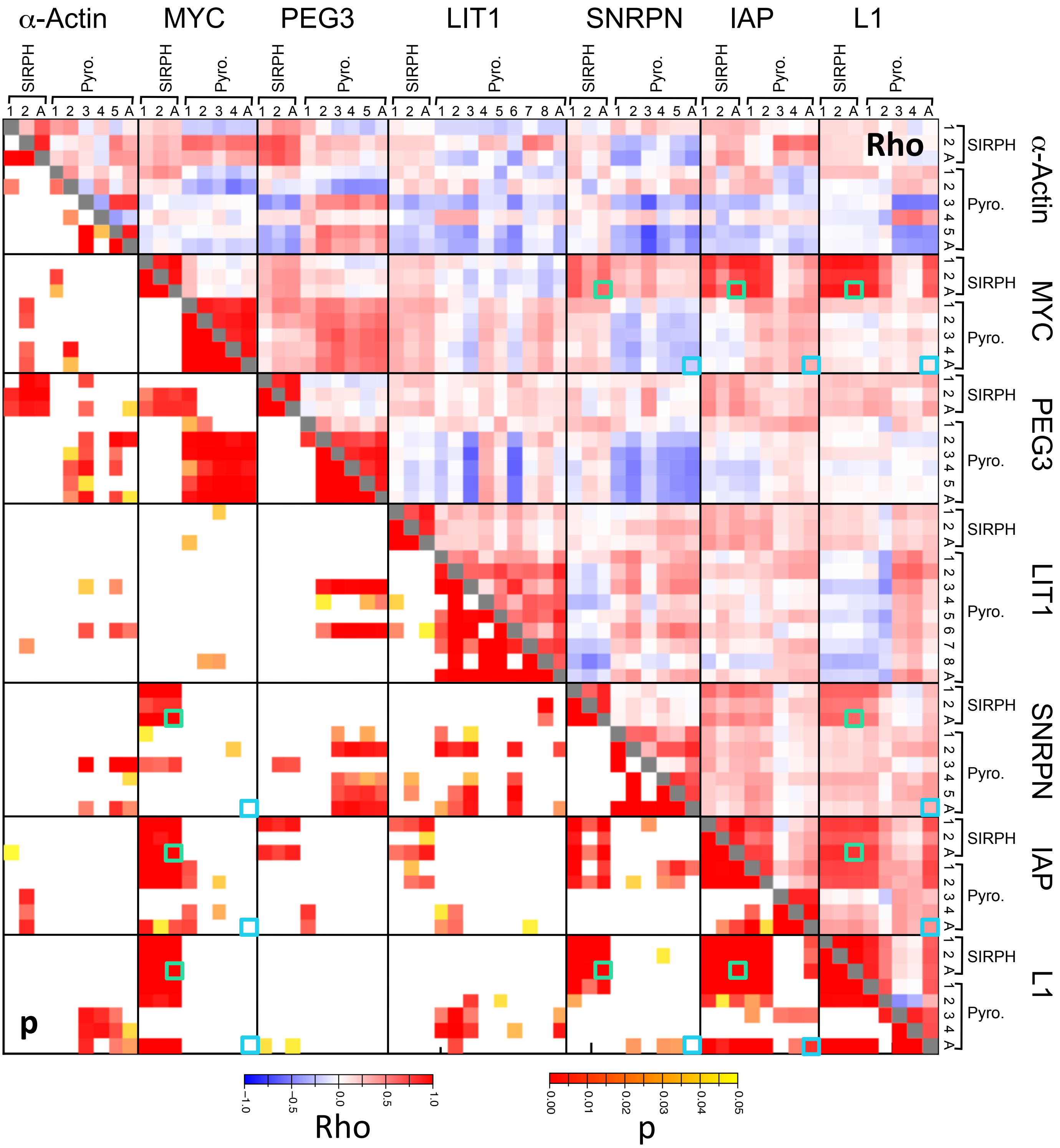

1, 2, …, A: CpG-1, CpG-2, etc, and average;
note that the CpG numbers for SIRPH and Pyro. do not necessarily correspond to the same CpG.

	α–Actin	α–Actin	MYC	MYC	PEG3	LIT1	SNRPN	IAP	IAP	L1
**SIRPH**	1	2	1	2	1	1	1	1	2	1
**Pyro**	1	2	1	3	1	1	1	1	3	1
**Rho**	0,37	0,08	0,25	0,08	-0,08	0,13	0,02	0,71	0,02	0,88
**P**	5,51E-02	7,86E-01	1,55E-01	6,45E-01	6,60E-01	4,57E-01	1,29E-01	5,44E-07	9,14E-01	1,34E-12
